# The Selective DHCR24 Blocker SH42 Inhibits ACE2 Binding and Cellular Entry of SARS-CoV-2 Spike Proteins More Efficiently Than Atorvastatin

**DOI:** 10.34133/research.1280

**Published:** 2026-05-14

**Authors:** Tamas Kovacs, Kitti Kurtan, Bernadett Pályi, Zoltán Kis, Mohamed Mahdi, Jesús Borrego, József Tőzsér, Zoltan Varga, Peter Nagy, Gyorgy Panyi, Florina Zakany

**Affiliations:** ^1^ Department of Biophysics and Cell Biology, Faculty of Medicine, University of Debrecen, Debrecen, Hungary.; ^2^ National Biosafety Laboratory, National Center for Public Health and Pharmacy, Budapest, Hungary.; ^3^ Institute of Medical Microbiology, Faculty of Medicine, Semmelweis University, Budapest, Hungary.; ^4^ Department of Biochemistry and Molecular Biology, Faculty of Medicine, University of Debrecen, Debrecen, Hungary.

## Abstract

ACE2 binding of spike proteins and concomitant viral uptake are the first and most decisive membrane-coupled events of SARS-CoV-2 infection, which rely on cholesterol-rich lipid raft microdomains of the host cell plasma membrane. Therefore, lowering membrane cholesterol levels may combat the infection, potentially complementing specific, resistance-prone antiviral approaches such as vaccines. High-throughput flow cytometry and quantitative 3-dimensional microscopy reveal that SH42, a novel, extremely potent, and highly selective 24-dehydrocholesterol reductase (DHCR24) blocker, markedly inhibits ACE2 binding of SARS-CoV-2 spike receptor-binding domains and entry of spike trimers of Wuhan-Hu-1 (WT), Delta, and Omicron BA.1 variants into living cells. These effects are related to SH42-induced reduction of plasma membrane cholesterol abundance and subsequent lipid raft disruption, which are accompanied by decreased cell surface ACE2 expression and its lowered raft partitioning. SH42 exhibits superior efficacy and potency in all aspects compared to the reference atorvastatin. Furthermore, inhibitory effects of SH42 are also corroborated by reduced SARS-CoV-2 RNA copy numbers of replication-competent complete virions of WT-resembling D614G, Delta, and Omicron-derived JN.1 strains. These results suggest that selective DHCR24 blockers such as SH42 may be promising novel candidates for inhibiting initial membrane-coupled events of SARS-CoV-2 infection and serve as potential alternative therapeutic option for COVID-19.

## Introduction

Severe acute respiratory syndrome coronavirus 2 (SARS-CoV-2), the pathogen of the coronavirus disease 2019 (COVID-19) pandemic, belongs to *Coronaviridae*, a family of enveloped, positive-strand RNA viruses that are characteristically decorated by surface spike glycoproteins [1]. The cellular invasion mechanism of SARS-CoV-2 is initiated by binding of the receptor-binding domain (RBD) on the S1 subunit of its spike to a receptor in the plasma membrane of the target cell, predominantly angiotensin-converting enzyme 2 (ACE2) [2,3]. This is followed by the proteolytic cleavage at the S2′ site of the spike that is mainly performed by transmembrane protease serine 2 (TMPRSS2) in the plasma membrane of lower respiratory tract cells or, particularly in cells with low TMPRSS2 expression such as upper airway tissues, cathepsin L in endolysosomes after a receptor-mediated endocytic uptake of the virus particle. These lead to shedding of S1 from the S2 subunit enabling membrane fusion, which finally terminates in the release of viral RNA into the cytoplasm of the infected cell [4–8]. The ACE2 binding of spike RBD and cellular entry of viral particles as the main early membrane-coupled steps of infection are substantially influenced by membrane cholesterol for several reasons. First, cholesterol-dependent ACE2 partitioning into lipid raft microdomains of the host cell facilitates spike binding and its localization in the vicinity of TMPRSS2 for cleavage [9–11]. Furthermore, endocytic processes, including lipid raft-mediated and clathrin-dependent pathways, intrinsically depend on membrane cholesterol [4,5,12,13].

In spite of tremendous efforts to develop therapeutic strategies and their relative success, COVID-19 still represents a major worldwide health concern and a potential ticking health bomb since broad-spectrum antiviral tools generally exhibit low selectivity and potentially poor safety profiles, whereas highly selective approaches including vaccines may be prone to resistance owing to continuously emerging variants [14,15]. While these SARS-CoV-2 variants, including the clinically relevant original Wuhan-Hu-1 strain (wild-type [WT]), B.1.617.2 (Delta), B.1.1.529 (Omicron BA.1), and BA.2.86.1.1 (JN.1), mainly differ in the amino acid sequence of the spike RBD and show different susceptibility to antibody neutralization and immune escape [16,17], the cellular uptake mechanisms in general, and their dependence on membrane cholesterol in particular, seem to be conserved according to literature data [5,9,18]. In accordance with these substantial roles of cholesterol in cellular entry, reduction of membrane cholesterol levels and concomitant lipid raft disruption may exert beneficial actions in the prevention and adjuvant treatment of COVID-19 by interfering with the early membrane-coupled steps of cellular infection [13,19,20].

In our previous study, we demonstrated using different SARS-CoV-2 spike variants in Calu-3 human lung adenocarcinoma cells that cyclodextrins, well-tolerable cyclic oligosaccharides such as those used in Veklury formulations to solubilize remdesivir, capable of depleting cholesterol from host cell membranes, reduce cellular binding and entry of SARS-CoV-2 spikes of WT, Delta, and Omicron BA.1 variants, and thus provide notable additive effects to the antiviral remdesivir even at formulational doses [9]. In addition, statins, blockers of 3-hydroxy-3-methyl-glutaryl-coenzyme A (HMG-CoA) reductase and the most common agents to lower cholesterol levels in human medicine, similarly inhibited events of SARS-CoV-2 infection in in vitro and in vivo models [10,21] and were also proposed to reduce COVID-19 mortality in patients [22]. However, certain epidemiological studies questioned the efficacy of de novo initiated statin therapy [23]. This controversy may arise from their limited membrane cholesterol-reducing efficiency or, alternatively, low doses that were applied to avoid potential severe side effects arising due to blocking de novo cholesterol biosynthesis at a proximal step [24], which points at the urging need of more efficient and tolerable statin alternatives. Δ24-Dehydrocholesterol reductase (DHCR24) is the terminal rate-limiting enzyme of cholesterol biosynthesis catalyzing the transformation of desmosterol to cholesterol, and its inhibition shows promise in various diseases associated with abnormal sterol levels including cardiovascular and metabolic disorders, Alzheimer’s disease, prostate cancer, and hepatitis C infection [25]. SH42 is a newly designed, extremely potent, and highly selective steroidal DHCR24 inhibitor [26], which exhibited good safety profiles and high efficiency to reduce total cellular cholesterol accumulation and induce beneficial metabolic changes. It also attenuated inflammation in cellular and animal models of murine peritonitis and diet-induced human-like nonalcoholic steatohepatitis in the absence of typical statin-related adverse effects such as liver damage or rhabdomyolysis [27,28]. However, its applicability has not yet been tested in SARS-CoV-2 in spite of the proposed better tolerability and improved efficacy regarding cholesterol reduction compared to statins.

In this work, our objective was to demonstrate the effects of DHCR24 inhibition with a novel selective blocker, SH42, on the initial host cell membrane-coupled events of SARS-CoV-2 infection, in comparison with atorvastatin (ATO), a well-established cholesterol-lowering drug in human medicine. In living cells, we found by flow cytometry that SH42 reduced total plasma membrane cholesterol levels and, as demonstrated by quantitative confocal microscopy, lowered cholesterol abundance in raft regions of the cell membrane, and disrupted their integrity along with extensively reducing their relative area in the membrane more efficiently compared to ATO. Then, we combined the application of 2 widely utilized models of the cellular infection, HEK/ACE2 + TMPRSS2 cells with an exogenous overexpression of ACE2 and TMPRSS2 [29,30], and Calu-3 human lung adenocarcinoma cells with endogenous, physiological levels of these proteins [9,31,32], with our previously developed flow cytometry and 3-dimensional (3D) quantitative microscopy assays to exclusively study the ACE2 binding and cellular entry of viral spike proteins [9]. As shown by flow cytometry experiments with WT, Delta, and Omicron BA.1 spike RBDs, SH42 induced dramatic decreases in ACE2 binding of all 3 examined RBD variants. Moreover, as shown by 3D quantitative microscopy, SH42 extensively inhibited intracellular accumulation of WT, Delta, and Omicron BA.1 spike trimers. Our further experiments revealed that SH42 may exert these actions by lowering cell surface ACE2 expression and decreasing ACE2 partitioning into lipid raft microdomains, thereby lowering the amount of accessible gates appropriate for the viral entry. Furthermore, SH42 effectively reduced viral RNA copy numbers in Vero cells infected with replication-competent complete virions including WT-based viruses carrying the D614G spike mutation, as well as the Delta, and the Omicron-derived JN.1 variants. While ATO treatment also resulted in such effects, the effects of SH42 were superior to that of ATO in all the examined parameters, particularly at the physiologically more relevant lower nanomolar concentrations obtainable with conventional therapeutic protocols in the serum, as well as at micromolar levels typically applied in cellular studies. Notably, changes elicited by 10 nM SH42 in most cases were statistically indistinguishable from that exerted by 1 μM ATO, indicating an orders-of-magnitude larger potency of SH42. Altogether, our data demonstrate more than 40% decreases in ACE2 binding, higher than 50% reductions in cellular uptake, and decreases in viral RNA copy numbers of replication-competent SARS-CoV-2 virions across all examined variants in response to SH42. These results suggest that DHCR24 inhibitors, especially SH42, can be promising candidates to inhibit initial membrane-coupled events of SARS-CoV-2 infection and, consequently, to decrease viral infection effectively by interfering with the cholesterol-dependent mechanisms utilized by the virus.

## Results

### The DHCR24 inhibitor SH42 decreases plasma membrane cholesterol abundance and leads to disruption of lipid rafts more efficiently than ATO

**Fig. 1.**
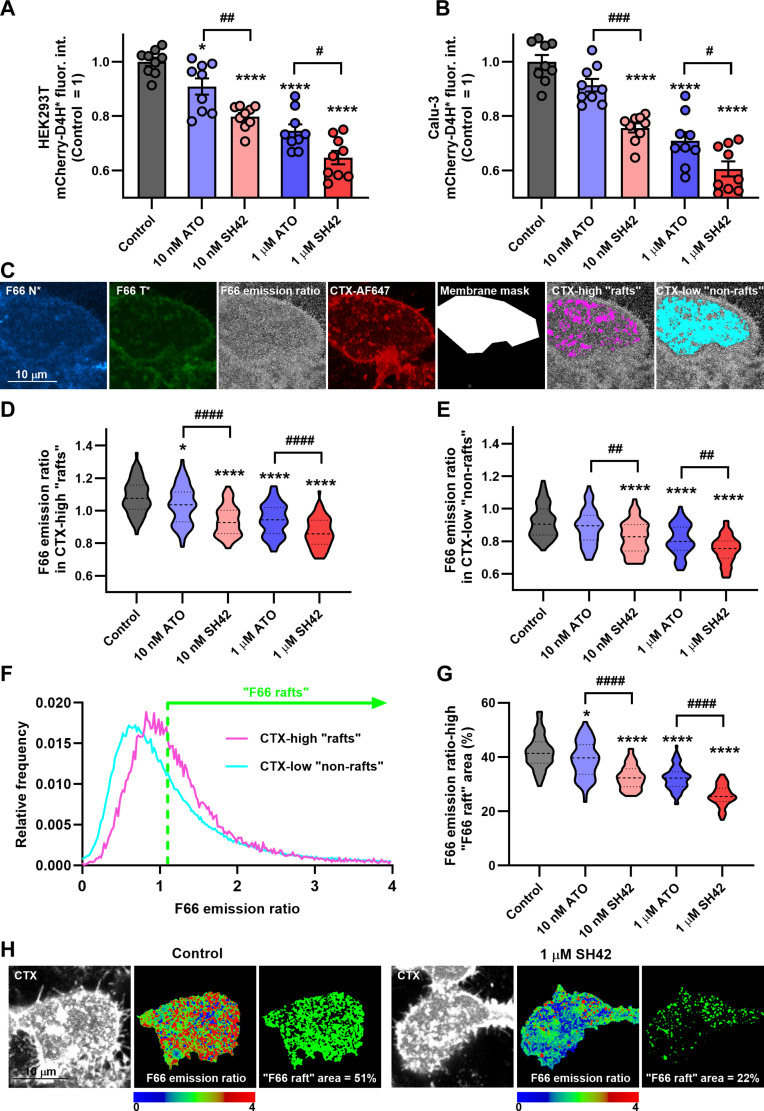
SH42 reduces cholesterol abundance in the plasma membrane in general and lipid rafts in particular, and decreases lipid raft area more efficiently than atorvastatin (ATO). Control HEK293T (A) and Calu-3 (B) cells and those treated for 96 h with 10 nM or 1 μM ATO or SH42 were labeled with cholesterol-binding mCherry-conjugated D4H*, the D434S mutant of domain 4 (D4) of *C. perfringens* theta-toxin. Fluorescence intensities correlating with plasma membrane cholesterol levels of at least 10,000 individual cells per sample were subsequently measured using a flow cytometer. The average intensity values obtained in *n* = 9 independent biological replicates, and their average values (± SEM) are plotted in the figure. (C) To examine changes in the cholesterol content of raft and non-raft microdomains of the plasma membrane, control HEK/ACE2 + TMPRSS2 cells and those treated as above were labeled with Alexa Fluor 647-conjugated cholera toxin subunit B (CTX-AF647), a lipid raft marker, and F66. F66 is a fluorescent indicator with spectral properties depending on the cholesterol-dependent local molecular order (dipole potential) of the membrane; therefore, this dye, combined with CTX-AF647, can provide information about the extent of cholesterol reduction separately in raft and non-raft membrane regions. Representative confocal microscopic images taken from the flat, bottom membrane region adjacent to the coverglass show F66 intensities detected in 2 wavelength ranges of emission (“F66 N*” and “F66 T*”), their ratio (“F66 emission ratio” calculated as T*/N* pixel by pixel), and CTX-AF647 intensities. Cell “membrane masks” selected manually in CTX images were segmented using the maxentropy algorithm to CTX-high “rafts” and CTX-low “non-rafts” corresponding to high- and low-intensity regions, respectively, as shown by the representative images. Violin plots were generated from median F66 emission ratio values determined separately for the CTX-high “raft” (D) and CTX-low “non-raft” (E) masks of *n* = 54 to 73 individual cells, which also display median values with quartiles. (F) Pixelwise distributions of the F66 emission ratio in CTX-high “rafts” and CTX-low “non-rafts” of control cells are displayed. For the quantification of the relative area of lipid rafts, as an alternative definition for raft regions, a threshold value of the F66 emission ratio was determined (green dashed line) and membrane pixels were considered as “F66 raft” and “F66 non-raft” regions when being above and below the threshold, respectively. (G) Violin plots were generated from the relative fraction of F66 raft pixels (“F66 raft area”) of individual cells, which also display median values with quartiles. (H) Representative images show changes in the lateral distribution of the F66 emission ratio on a color-scale image and reduction in the relative F66 raft area induced by 1 μM SH42. Throughout the figure, asterisks indicate significant differences compared to control samples (*P < 0.05, ****P < 0.0001), while hashes show that between samples treated with ATO and SH42 at identical concentrations (^#^*P* < 0.05, ^##^*P* < 0.01, ^###^*P* < 0.001, ^####^*P* < 0.0001), which were determined by Tukey’s HSD test carried out after significant differences were obtained for between-group effects in ANOVA.

Because no data are available on how SH42 affects plasma membrane cholesterol level—and because cholesterol-rich microdomains are crucial for the early membrane-coupled steps of SARS-CoV-2 infection [9–11,13]—we first compared the effects of SH42 with those of ATO on plasma membrane cholesterol content and lipid raft integrity. We applied the compounds at 10 nM mimicking statin concentrations that are expected in clinical protocols and at 1 μM concentration that is typically applied in in vitro studies with statins [33]. We examined the effects of 4-d SH42 and ATO treatments on the plasma membrane cholesterol levels of individual cells using flow cytometry with mCherry-conjugated D4H*, the D434S mutant of domain 4 (D4) of *Clostridium perfringens* theta-toxin. This peptide selectively binds cholesterol molecules in the plasma membrane and has been successfully applied to follow changes in plasma membrane cholesterol content in living cells [34,35]. In these measurements, while both SH42 and ATO reduced plasma membrane cholesterol levels in a concentration-dependent manner in HEK293T and Calu-3 cells, SH42-induced reductions were significantly higher than that of ATO at both examined concentrations (Fig. 1A and B and Fig. S2). In control experiments, we showed that neither SH42 nor ATO altered the fraction of viable cells according to a flow cytometer-based method (Fig. S1).

Given that cholesterol is present in both raft and non-raft microdomains of the plasma membrane, we compared the extents of cholesterol reduction in raft and non-raft membrane regions in HEK/ACE2 + TMPRSS2 cells, a standard model of in vitro SARS-CoV-2 research [29,30]. For this, we used F66, an environment-sensitive fluorescent dye responsive to membrane order through its sensitivity to the dipole potential, an order-related membrane biophysical parameter [36–38]. Cholesterol content and thus both membrane order and the dipole potential are larger in lipid rafts than in non-raft domains [9,36]. Furthermore, the emission ratiometric measurement of the spectral shift of F66 reliably reports on the cholesterol abundance due to its strong correlation with membrane order and the dipole potential. We labeled cells with F66 and Alexa Fluor 647-conjugated cholera toxin subunit B (CTX) that selectively binds to raft-resident GM1 ganglioside and thus visualizes lipid raft microdomains. Subsequently, we acquired images from the flat, bottom membrane regions of cells adjacent to the coverglass. During image analysis, we manually selected the regions corresponding to the cell membrane and segmented the images into raft and non-raft regions based on the CTX fluorescence intensity of membrane pixels (Fig. 1C). We separately characterized F66 emission ratio, that is, membrane order (dipole potential), in the CTX-high “raft” and CTX-low “non-raft” areas to detect cholesterol-related alterations in the 2 membrane regions. SH42 led to remarkable dose-dependent reductions in the dipole potential measured by the spectral shift of F66 in both CTX-high “raft” and CTX-low “non-raft” areas (Fig. 1D and E). Again, although ATO elicited similar effects, those induced by SH42 were significantly larger in both membrane regions at both examined concentrations. In fact, changes in response to 10 nM SH42 and 1 μM ATO were indistinguishable. These results suggested that membrane cholesterol reduction in response to SH42 and, to a lower extent, ATO is mirrored by a modified membrane structure involving not only non-raft but raft microdomains of the cell membrane as well; thus, cholesterol reduction induced by these compounds can be expected to interfere with the raft-dependent entry of SARS-CoV-2.

Consistent with our previous results [9,36], the average F66 emission ratios were systematically higher in CTX-high domains than outside them in all treatment conditions (Fig. 1D and E). In accordance, an alternative analysis method can be performed to estimate the relative area occupied by lipid raft regions by defining these microdomains based on a high magnitude of dipole potential in individual cells. With this method, we can follow changes in the integrity of lipid rafts that is a crucial determinant of viral entry. For this, we compared the F66 emission ratio histograms of CTX-high and CTX-low areas of control samples and determined a threshold value (Fig. 1F), which was chosen to result in a segmentation pattern similar to that obtained in the first method. Subsequently, we defined the pixels as “F66 rafts” when having an F66 emission ratio above the threshold, and calculated the fraction of “F66 rafts” compared to the total membrane area for each individual cell. We observed strongly consistent results, as SH42 notably and concentration-dependently reduced the relative area occupied by “F66 rafts” that was approximately 42% in control cells and less than 26% in cells treated with 1 μM SH42. SH42 elicited significantly larger effects than ATO at both concentrations, and again, effects induced by 10 nM SH42 and 1 μM ATO were statistically identical (Fig. 1G and H). Overall, these findings support that SH42 extensively alters the integrity of lipid raft microdomains of the plasma membrane, thereby possibly reducing the amount of accessible viral entry points required for cellular infection.

### The DHCR24 inhibitor SH42 reduces ACE2 binding of SARS-CoV-2 spike RBDs more efficiently than ATO

**Fig. 2.**
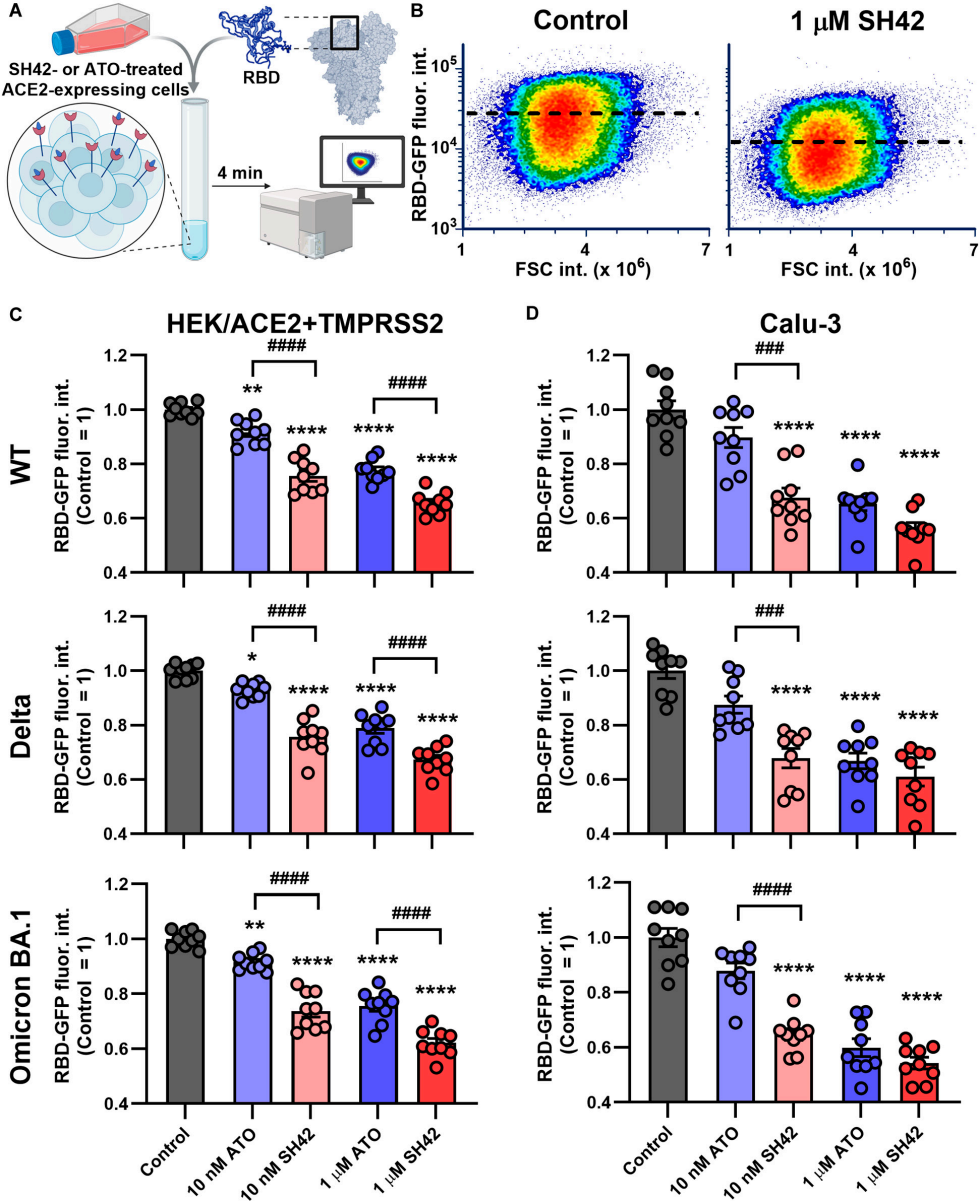
SH42 decreases ACE2 binding of SARS-CoV-2 spike receptor-binding domains (RBDs) more efficiently than ATO. (A) ACE2-expressing HEK/ACE2 + TMPRSS2 and Calu-3 control cells and those treated for 96 h with 10 nM or 1 μM ATO or SH42 were incubated with the GFP-conjugated RBDs of the Wuhan-Hu-1 strain (WT), Delta, and Omicron BA.1 variants for 4 min. RBDs were applied at 0.2 and 1.0 μg/ml for HEK/ACE2 + TMPRSS2 and Calu-3 cells, respectively. Fluorescence intensities of at least 10,000 individual cells per sample were subsequently measured using a flow cytometer. (B) Representative RBD-GFP versus forward-scattered light intensity (FSC) density plots demonstrate decreases in the bound WT RBD-GFP in response to 1 μM SH42 in HEK/ACE2 + TMPRSS2 cells. Dashed lines represent average values of the fluorescence intensity obtained in the displayed representative samples. The average intensities obtained in *n* = 9 independent biological replicates and normalized to the mean value determined in untreated control samples, and their average values (± SEM) are plotted for WT, Delta, and Omicron BA.1 variants in HEK/ACE2 + TMPRSS2 (C) and Calu-3 (D) cells. Throughout the figure, asterisks indicate significant differences compared to control samples (*P < 0.05, **P < 0.01, ****P < 0.0001), while hashes show those between samples treated with ATO and SH42 at identical concentrations (^###^*P* < 0.001, ^####^*P* < 0.0001), which were determined by Tukey’s HSD test carried out after significant differences were obtained for between-group effects in ANOVA.

Since we [9] as well as others [10,21] have demonstrated recently that membrane cholesterol depletion and consequent lipid raft disruption interfere with the first and most decisive step of interaction of SARS-CoV-2 with target cells, that is, RBD binding to the main receptor ACE2, we studied the effects of SH42 on this process. For this, we applied a flow cytometer-based method utilizing green fluorescent protein (GFP)-conjugated RBDs, which we recently optimized to quantify RBD binding to the surface of target cells (Fig. 2A). This high-throughput method is capable of following specific RBD binding in a sensitive and strongly quantitative manner due to the 1:1 RBD–fluorophore ratio [9]. We examined 3 variants, WT, Delta, and Omicron BA.1, that carry mutations in the RBD [16,17], and performed measurements in HEK/ACE2 + TMPRSS2 cells, a commonly utilized cellular model for SARS-CoV-2 infection in spite of having supraphysiological amounts of ACE2 [29,30], and Calu-3 cells that express ACE2 endogenously at lower physiological levels [9,31,32] to exclusively study the initial membrane-coupled event of cellular infection, the receptor–ligand interaction. After a 4-min incubation in the presence of RBDs when, as we showed previously, cell surface binding of RBDs is dominant and the early variable phase of receptor–ligand interaction is over [9], a considerable amount of cellular binding was found in both cell lines. In Calu-3 cells, even at the higher applied RBD concentrations, consistent with our expectations, much lower binding was found (Fig. S3). In our representative measurements, 1 μM SH42 drastically reduced cell-bound WT RBD-GFP without affecting cell size or morphology (Fig. 2B). Our detailed analysis of HEK/ACE2 + TMPRSS2 cells showed that both SH42 and ATO significantly reduced cell-bound fluorescence signals, indicating an inhibition of binding of all 3 examined variants in a concentration-dependent manner. The extents of reduction were significantly larger in response to SH42 when compared to that of ATO at both tested concentrations, reaching a maximum magnitude of approximately 33% to 38% with 1 μM SH42 (Fig. 2C). In fact, decreases elicited by 10 nM SH42 were statistically indistinguishable from that of 1 μM ATO. Similar results were found in Calu-3 cells, since both treatments concentration-dependently inhibited binding of all 3 examined RBDs (Fig. 2D). In general, in accordance with our previous findings (*9*), reductions were slightly larger in Calu-3 cells than in HEK/ACE2 + TMPRSS2, and the decreases were approximately 39% to 46% in response to 1 μM SH42. Additionally, differences between the efficiencies of SH42 and ATO were more obvious at 10 nM. For example, in the WT variant, SH42 and ATO resulted in decreases of 24% and 8% at 10 nM, and 34% and 22% at 1 μM, respectively, in HEK/ACE2 + TMPRSS2 cells, and 33% and 10% at 10 nM, and 44% and 35% at 1 μM, respectively, in Calu-3 cells. As seen in our previous study (*9*), decreased membrane cholesterol-induced inhibition was slightly smaller in the case of the Delta variant when compared to WT, whereas the largest effects were seen with Omicron BA.1. Nevertheless, SH42 effectively reduced RBD binding in all variants.

**Fig. 3.**
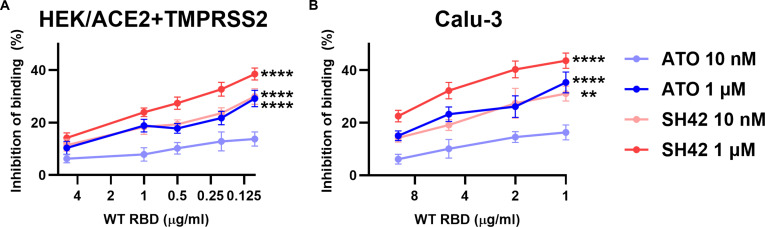
SH42-induced reduction in ACE2 binding of WT SARS-CoV-2 spike RBDs negatively correlates with the applied RBD concentration. ACE2-expressing HEK/ACE2 + TMPRSS2 (A) and Calu-3 (B) control cells and those treated for 96 h with 10 nM or 1 μM ATO or SH42 were incubated with different concentrations of the GFP-conjugated RBDs of the Wuhan-Hu-1 strain (WT) for 4 min. Fluorescence intensities of at least 10,000 individual cells per sample were subsequently measured using a flow cytometer. The extents of inhibition (calculated as 1 − average of treated/average of control) were determined in *n* = 9 independent biological replicates, and their average values (± SEM) are plotted as a function of the applied RBD concentration ranging between 0.1 and 5 μg/ml for HEK/ACE2 + TMPRSS2 and between 1 and 10 μg/ml for Calu-3 cells. Asterisks indicate significant differences between samples treated with the lowest versus highest RBD concentrations for each treatment (**P < 0.01, ****P < 0.0001), which were determined by Tukey’s HSD test carried out after significant differences were obtained for between-group effects in ANOVA.

In this flow cytometer-based binding assay, while we tried to minimize the amounts of RBD used, due to technical limitations of quantifying fluorescence, supraphysiological concentrations of RBD had to be applied. However, as we and others demonstrated previously, one cell is infected typically by one virion under pathophysiological conditions [9,39]. Therefore, we quantified the extent of inhibition of RBD binding by SH42 by applying WT RBD-GFP at different concentrations. We observed a negative correlation between the utilized RBD concentration and the extent of SH42-induced reduction in binding in both HEK/ACE2 + TMPRSS2 and Calu-3 cells (Fig. 3A and B, respectively), implying that even larger effects could be expected at low pathophysiologically relevant RBD concentrations. A similar tendency was revealed with ATO as well.

### The DHCR24 inhibitor SH42 decreases cellular entry of SARS-CoV-2 spike trimers more efficiently than ATO

**Fig. 4.**
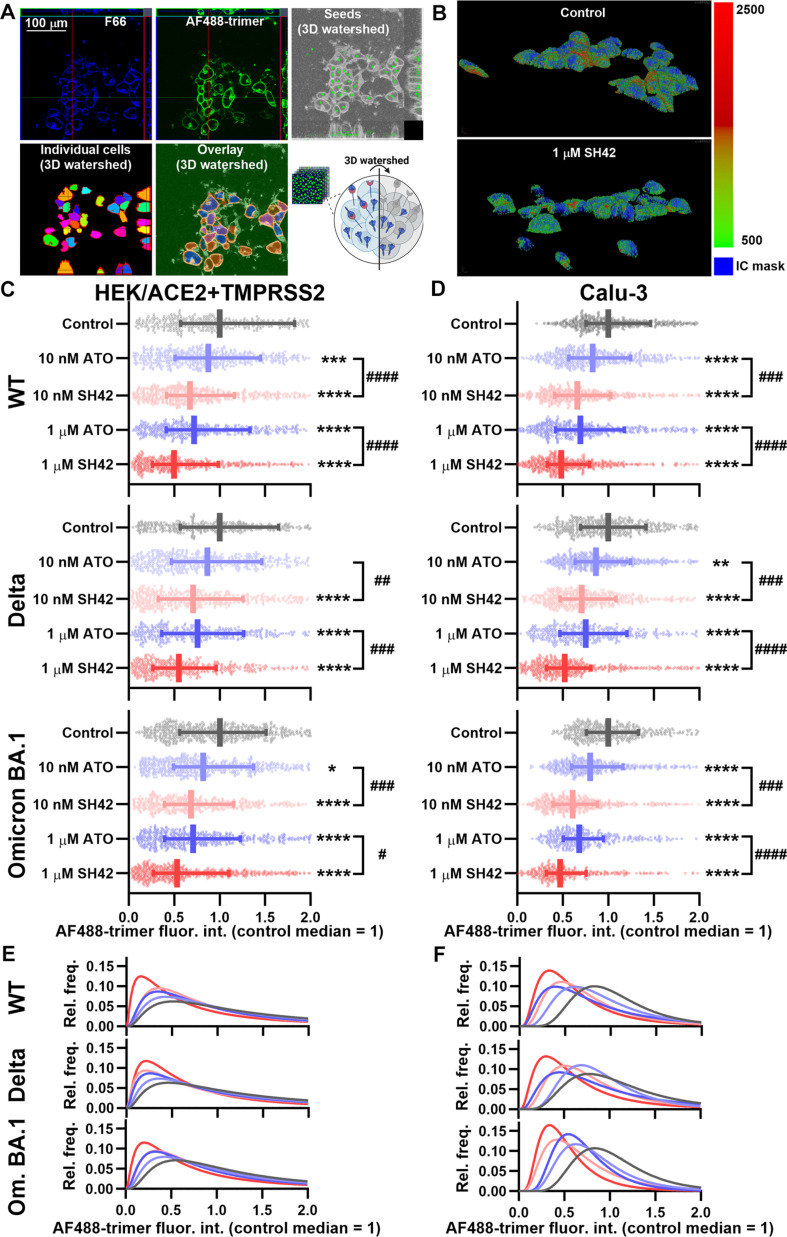
SH42 inhibits the cellular entry of SARS-CoV-2 spike trimers more efficiently than ATO. Control HEK/ACE2 + TMPRSS2 and Calu-3 cells and those treated for 96 h with 10 nM or 1 μM ATO or SH42 were incubated for 4 h in the presence of WT, Delta, or Omicron BA.1 SARS-CoV-2 spike trimers conjugated with Alexa Fluor 488 (AF488-trimers) and labeled with F66. (A) Representative orthogonal views of confocal Z-stack images of F66 for the visualization of the plasma membrane and AF488-trimers to estimate entry demonstrate notable trimer accumulation in the intracellular space of untreated control HEK/ACE2 + TMPRSS2 cells. During image analysis, pixels corresponding to plasma membrane and intracellular pixels were segmented based on F66 Z-stack images. Markers were manually placed inside cells (green circles in the grayscale orthogonal view), and a MATLAB implementation of the 3D watershed algorithm identified the intracellular space of cells and their membrane (colored regions and red lines in the orthogonal view in the middle, respectively, and their overlay image displayed on the right). (B) Representative 3D reconstruction images displaying AF488 fluorescence intensities on a green-red color scale above a threshold intensity overlaid on intracellular pixels of individual cells (in transparent blue) demonstrate decreases in the amount of intracellular WT trimers in response to 1 μM SH42. Subsequently, the average fluorescence intensity values emitted by AF488-trimers were calculated exclusively from data of intracellular pixels for individual cells. The average intensities obtained in *n* = 400 to 600 HEK/ACE2 + TMPRSS2 (C) and Calu-3 (D) cells and normalized to the median value determined in untreated control samples are plotted along with median values with quartiles for WT, Delta, and Omicron BA.1 trimer variants. Asterisks indicate significant differences compared to control samples (*P < 0.05, **P < 0.01, ***P < 0.001, ****P < 0.0001), while hashes show that between samples treated with ATO and SH42 at identical concentrations (^#^*P* < 0.05, ^##^*P* < 0.01, ^###^*P* < 0.001, ^####^*P* < 0.0001), which were determined by Tukey’s HSD test carried out after significant differences were obtained for between-group effects in ANOVA. Lognormal functions fitted to normalized mean intracellular AF488-trimer fluorescence intensity histograms of individual HEK/ACE2 + TMPRSS2 (E) and Calu-3 (F) cells also demonstrate the effects of ATO and SH42 on the internalization of WT, Delta, and Omicron BA.1 trimer variants.

Next, we investigated whether SH42-mediated cholesterol reductions and decreased ACE2 binding are mirrored in an inhibition of intracellular accumulation of SARS-CoV-2 spike trimers. We quantified these actions utilizing a confocal microscopy- and 3D quantitative image analysis-based approach that we optimized in our previous study [9], which provides information about the quantity of spike trimers entering the cells, irrespective of the exact entry mechanism, that is, cell surface entry mediated mainly by TMPRSS2 or endosomal entry. After a 4-d treatment, we incubated HEK/ACE2 + TMPRSS2 and Calu-3 cells with Alexa Fluor 488-conjugated WT, Delta, or Omicron BA.1 spike trimers for 4 h, and subsequently labeled them with F66 to visualize the plasma membrane unequivocally. Then, after acquiring confocal images with 20 to 25 slices, during quantitative image analysis, we identified plasma membranes of individual cells using a 3D watershed algorithm based on the F66 signal. Subsequently, we calculated the average Alexa Fluor 488 fluorescence intensity in only intracellular pixels of individual cells, which characterizes the total amount of trimers localized in the intracellular space without considering the ones at the cell surface (Fig. 4A). As demonstrated by the representative images of control HEK/ACE2 + TMPRSS2 cells and those treated with 1 μM SH42 (Fig. 4B), we observed notable effects on trimer entry as indicated by the strongly reduced fraction of red and green pixels displaying high intensities. Similarly to that found in experiments determining RBD binding, both SH42 and ATO decreased intracellular accumulation of all 3 examined spike trimers in HEK/ACE2 + TMPRSS2 cells in a concentration-dependent manner, and again, effects induced by SH42 were significantly larger than those of ATO, reaching an inhibition of approximately 45% to 50% with 1 μM SH42 (Fig. 4C). Measurements carried out in Calu-3 cells showed strongly consistent inhibition in response to SH42 and ATO, with SH42 being significantly more effective at 10 nM and leading to a reduction by approximately 47% to 52% at 1 μM (Fig. 4D). In general, just like in the case of RBD binding, differences between the efficiencies of SH42 and ATO were more obvious at 10 nM. For example, in the WT variant, SH42 and ATO resulted in decreases of 33% and 12% at 10 nM, and 50% and 28% at 1 μM, respectively, in HEK/ACE2 + TMPRSS2 cells, and 34% and 17% at 10 nM, and 52% and 30% at 1 μM, respectively, in Calu-3 cells. Additionally, changes induced by 1 μM ATO and 10 nM SH42 were indistinguishable in both cell types. These effects are also obvious in lognormal fits of normalized fluorescence intensity histograms of all 3 variants in both cell types (Fig. 4E and F). The alterations elicited by SH42 and ATO were generally slightly more pronounced in Calu-3 cells than in HEK/ACE2 + TMPRSS2. Furthermore, while a quantitative comparison is not unequivocal due to differences in measurement methods, in general, changes found in the trimer entry exceed those in RBD binding, which suggest that an inhibition of cellular internalization of spike trimers contributes to the actions of SH42 and ATO in addition to the reduced cell surface binding.

### The DHCR24 inhibitor SH42 reduces cell surface expression of ACE2 and its colocalization with lipid rafts more efficiently than ATO

**Fig. 5.**
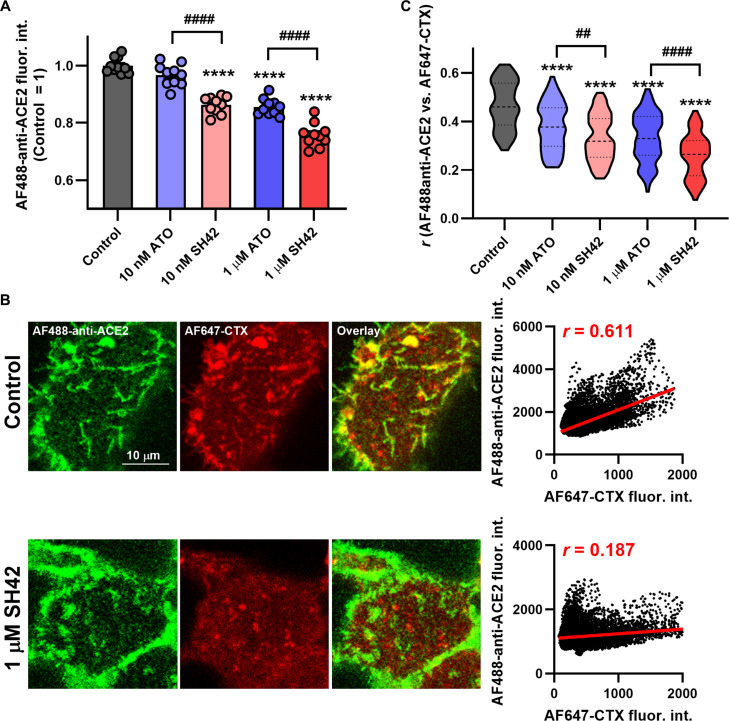
SH42 decreases cell surface ACE2 expression and its colocalization with lipid rafts more efficiently than ATO. (A) Control HEK/ACE2 + TMPRSS2 and Calu-3 cells and those treated for 96 h with 10 nM or 1 μM ATO or SH42 were labeled with Alexa Fluor 488-conjugated anti-ACE2 antibodies (AF488-anti-ACE2). Fluorescence intensities of at least 10,000 individual cells per sample were subsequently measured using a flow cytometer. The average intensities obtained in *n* = 10 independent biological replicates and normalized to the mean value determined in untreated control samples, and their average values (± SEM) are plotted in the panel. (B) Control cells and those treated as above were labeled with AF488-anti-ACE2 and Alexa Fluor 647-conjugated cholera toxin subunit B (AF647-CTX). Representative confocal microscopic images taken from the flat, bottom membrane region adjacent to the coverglass show AF488-anti-ACE2 and AF647-CTX intensities, and their overlay, while the colocalization of the 2 signals and its changes in response to 1 μM SH42 are displayed in representative dot plots obtained from pixelwise fluorescence intensities. (C) Violin plots were generated from Pearson correlation coefficient values between fluorescence intensities of the 2 applied fluorophores determined from pixelwise data of *n* = 81 to 90 individual cells, which also display median values with quartiles. Throughout the figure, asterisks indicate significant differences compared to control samples (*P < 0.05, **P < 0.01, ****P < 0.0001), while hashes show that between samples treated with ATO and SH42 at identical concentrations (^###^*P* < 0.001, ^####^*P* < 0.0001), which were determined by Tukey’s HSD test carried out after significant differences were obtained for between-group effects in ANOVA.

Next, we explored potential mechanisms behind the inhibited binding and internalization of SARS-CoV-2 spike proteins in response to SH42. The lipid raft localization can modify the stability and function of transmembrane proteins on the cell surface [40,41], and these microdomains are enriched in additional molecules required for viral entry such as TMPRSS2 or glycolipids [13,20]. Consistently, reduced membrane cholesterol levels and concomitantly decreased lipid raft abundance in response to cyclodextrins or statins have been previously shown to be associated with lower cell surface expression of ACE2 [9–11]. In accordance with these studies, when we estimated ACE2 amounts on the cell surface using flow cytometry and ACE2 antibodies, we found that both SH42 and ATO dose-dependently reduced ACE2 levels, with the former being significantly more effective (Fig. 5A and Fig. S4). ATO (1 μM) and SH42 resulted in a reduction of ACE2 expression of approximately 15% and 25%, respectively. These data support that the amount of membrane cholesterol positively correlates with the extent of ACE2 expression on the cell surface. However, the extents of reduction were notably smaller in plasma membrane ACE2 abundance than in either RBD binding or trimer internalization, which may refer to the contribution of additional factors.

The unique microenvironment provided by the lipid rafts leads to a cholesterol-dependent accumulation or segregation of membrane components, and consequently, disruption of these microdomains may alter the lateral distribution of certain transmembrane proteins [20,41]. For example, the preferential raft localization of ACE2 was found to be reduced by cyclodextrins capable of extracting cholesterol from the cell membrane [9,11]. Therefore, we examined the colocalization between ACE2 proteins visualized by antibodies and lipid rafts labeled with CTX using confocal microscopy and calculated the Pearson correlation coefficient quantifying the lipid raft partitioning of ACE2 (Fig. 5B). In control cells, we found strongly positive correlation coefficients (approximately 0.47 on average) that were far outside the 95% confidence intervals determined when assuming the absence of correlation falling in the range between −0.02 and 0.03. Both SH42 and ATO dose-dependently reduced these coefficients, mirroring a smaller raft accumulation of ACE2. In accordance with the effects on RBD uptake and trimer internalization, changes induced by SH42 were significantly larger than those of ATO, and those elicited by 1 μM ATO and 10 nM SH42 were quantitatively identical (Fig. 5C). Altogether, these results suggest that besides the reduced lipid raft and cell surface ACE2 abundance, the smaller preference of ACE2 toward the rafts also contributes to the ATO- and SH42-induced inhibition of SARS-CoV-2 spike binding and internalization.

### The DHCR24 inhibitor SH42 decreases RNA copy numbers of replication-competent SARS-CoV-2 virions more efficiently than ATO

**Fig. 6.**
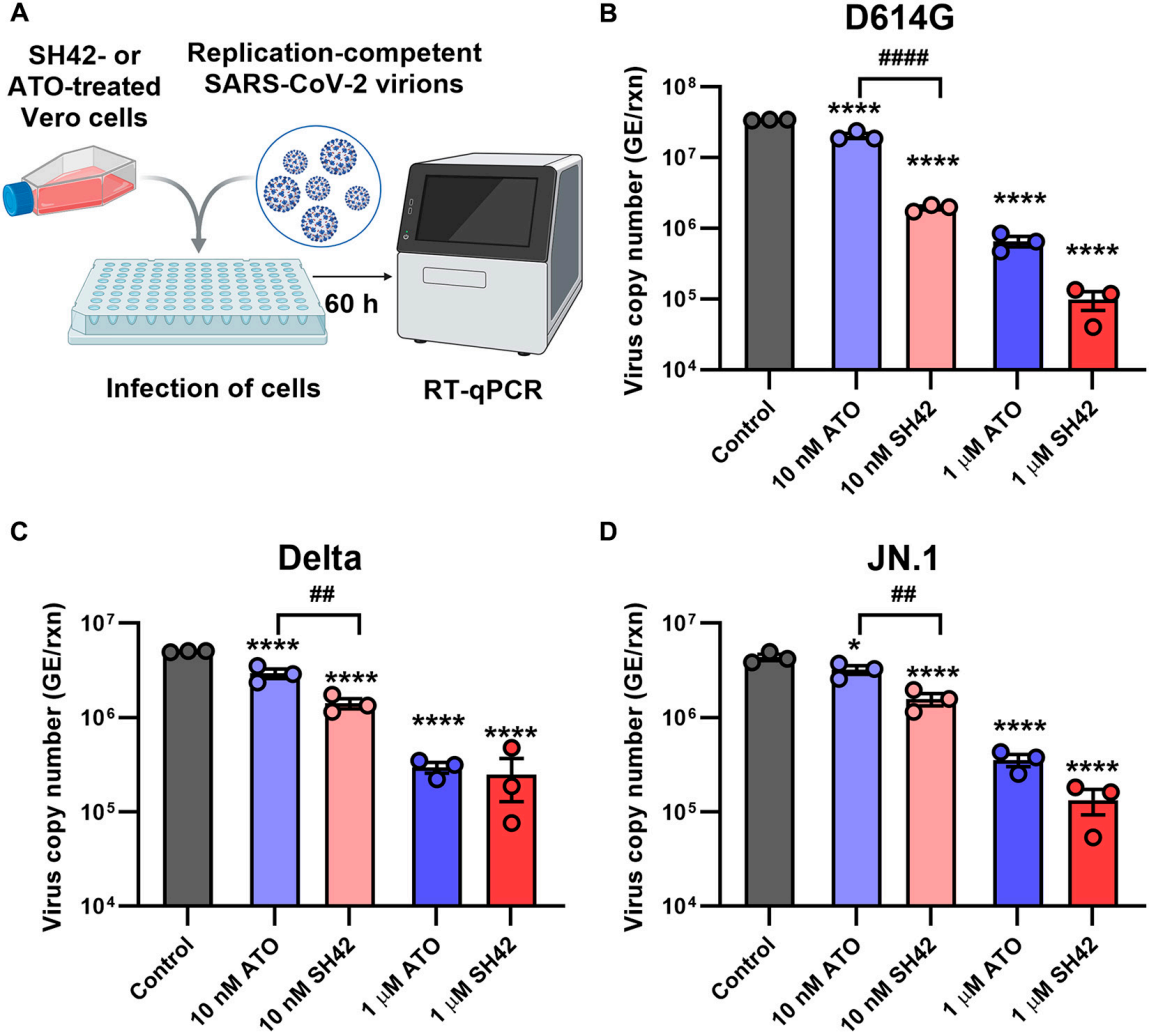
SH42 decreases RNA copy numbers of replication-competent SARS-CoV-2 virions more efficiently than ATO. (A) Control Vero cells and those treated for 96 h with 10 nM or 1 μM ATO or SH42 were seeded into 96-well plates and infected with WT-based virions carrying the D614G mutation, as well as Delta and JN.1 SARS-CoV-2 variants at 100 TCID_50_ per well. After 60 h, RNA was extracted from the collected supernatant and SARS-CoV-2 RNA copy numbers were determined by RT-qPCR using the CDC SARS-CoV-2 N1 primer/probe set. The viral RNA copy numbers obtained in *n* = 3 independent biological replicates, and their average values (± SEM) are plotted for D614G (B), Delta (C), and JN.1 (D) variants. Throughout the figure, asterisks indicate significant differences compared to control samples (*P < 0.05, ****P < 0.0001), while hashes show those between samples treated with ATO and SH42 at identical concentrations (^##^*P* < 0.01, ^####^*P* < 0.0001), which were determined by Tukey’s HSD test carried out after significant differences were obtained for between-group effects in ANOVA.

In the final part of the study, we aimed at further corroborating the potential relevance of DHCR24 inhibition against SARS-CoV-2. First, to investigate the entry process, we followed cellular binding of lentivirions pseudotyped with WT SARS-CoV-2 spike proteins, and found that SH42 decreased the transduction efficiency of HEK/ACE2 + TMPRSS2 cells, reflecting inhibited cellular uptake (Fig. S5). At 10 nM, SH42 induced a significantly larger decrease than the same concentration of ATO. Since our experiments with various models convincingly supported that DHCR24 blockade inhibits cellular entry, we examined whether these effects would also manifest in reduced cellular infection. Therefore, we investigated effects of the DHCR24 inhibitor SH42 in a cell-based infection assay with replication-competent complete SARS-CoV-2 virions (Fig. 6A). For these experiments, we utilized Vero cells in which we first confirmed that SH42 dose-dependently reduced plasma membrane cholesterol levels (Fig. S6A), WT RBD-GFP binding (Fig. S6B), and WT Alexa Fluor 488-conjugated spike trimer entry (Fig. S6C) just as in HEK/ACE2 + TMPRSS2 and Calu-3 cells. In the infection assay, Vero cells were pretreated for 96 h with ATO or SH42 and then infected with replication-competent complete virions and incubated for an additional 60 h with the appropriate treatment. Reverse transcription quantitative polymerase chain reaction (RT-qPCR) analysis of the supernatant collected from the cells revealed that SH42 significantly and dose-dependently decreased viral RNA copy numbers of 3 variants, including D614G closely resembling WT, Delta, and the Omicron-derived JN.1 (Fig. 6B, C and D, respectively). SH42 significantly outperformed ATO at 10 nM, and 1 μM SH42 was able to induce approximately 300-, 20-, and 30-fold reductions in the 3 variants, respectively. These results demonstrate that the SH42-induced inhibition of entry manifests in decreased copy numbers of the viral RNA after cellular infection and SH42 exhibits superior efficacy over ATO at 10 nM concentration, which support the anti-SARS-CoV-2 potential of the compound.

## Discussion

As the major finding of our study, we revealed previously unexplored effects of DHCR24 inhibition by using its extremely potent and highly selective steroidal blocker SH42. We showed that SH42 inhibits ACE2 binding and entry of viral spike proteins as the decisive, initial cholesterol-dependent membrane-coupled events of cellular SARS-CoV-2 infection, which in turn culminate in decreased viral RNA copy numbers after infecting cells with replication-competent virions. Considering its order-of-magnitude greater potency in the investigated parameters and more beneficial intended versus adverse effect profiles suggested by literature data [27,28] compared to that of ATO, a widely applied cholesterol-lowering statin, here we raise the possibility for the first time that DHCR24 blockers in general, and one of the most promising members of the family, SH42, in particular, can be a potential novel therapeutic approach against SARS-CoV-2.

**Fig. 7.**
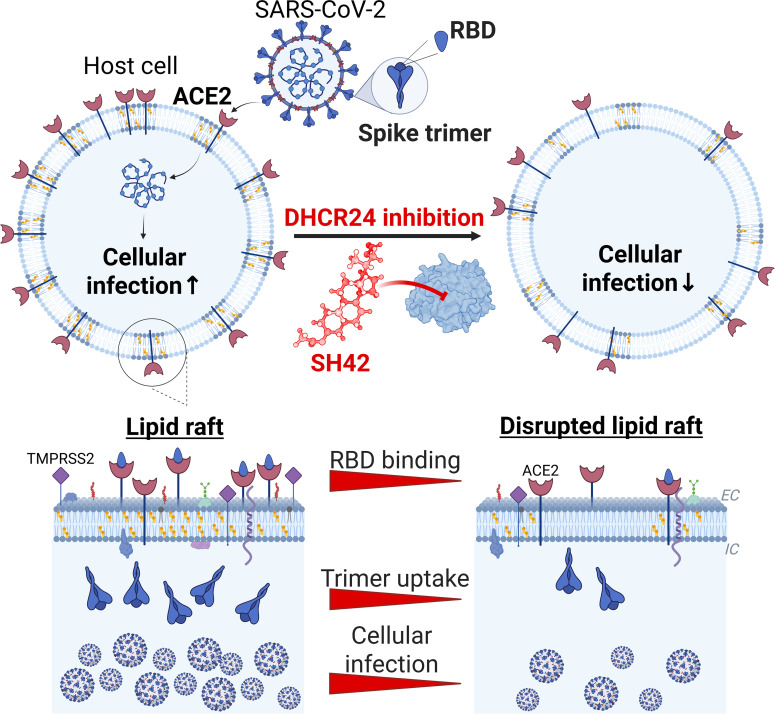
DHCR24 inhibition by its selective blocker SH42 is a potential novel therapeutic approach to suppress initial membrane-coupled events of SARS-CoV-2 infection. SH42, a novel steroidal highly selective and potent DHCR24 inhibitor, interferes with ACE2 binding of SARS-CoV-2 spike RBDs and cellular uptake of spike proteins. By efficiently decreasing cholesterol levels of the host cell plasma membrane and causing the concomitant disruption of lipid raft microdomains, SH42 decreases cell surface levels of ACE2 and, in addition, reduces raft partitioning of the receptor protein, thereby altering its local microenvironment required for an efficient ACE2-mediated cellular binding and uptake of the virus. As a result, early membrane-coupled events of SARS-CoV-2 infection are inhibited as mirrored by the decreased binding of spike RBDs to host membrane and decreased cellular uptake of spike trimers, and culminate in decreased cellular infection with replication-competent SARS-CoV-2 virions.

Initial steps of cellular invasion of SARS-CoV-2 include ACE2 binding of the viral spike glycoprotein and its cleavage by cell surface proteases, mainly TMPRSS2, or lysosomal cathepsin L after endocytosis [4,5,8], and all of these events are inhibited by SH42. Namely, according to flow cytometry measurements, SH42 dose-dependently and notably reduced ACE2 binding of spike RBDs, the reduction reaching approximately 40% in response to 1 μM SH42 (Fig. 2 and Fig S3). Furthermore, as we determined with 3D confocal microscopy, SH42 decreased total intracellular accumulation of spike trimers (Fig. 4). Remarkably, these changes were slightly but consistently larger than those observed in RBD binding exceeding 50% with 1 μM SH42. Our 3D microscopy-based analysis quantified intracellular accumulation of spike trimers irrespective of the exact entry mechanisms, and we did not differentiate between cell surface and endosomal entry. However, our strongly consistent results obtained in cell lines with distinct TMPRSS2 expression and consequently different utilization of the 2 pathways might imply that SH42 could interfere with both major entry routes. These inhibitory effects appeared to be conserved in different variants and host cell types, as similar results were observed with the WT, Delta, and Omicron BA.1 variants in HEK/ACE2 + TMPRSS2 and Calu-3 cells, which express different levels of ACE2 [9,29–32], as well as with the WT spike in Vero cells, which lack notable TMPRSS2 expression [42,43]. These SH42 effects are also reflected by notably decreased viral RNA copy numbers in Vero cells infected with replication-competent complete virions of variants including D614G resembling WT, Delta, and the Omicron-descendant JN.1 prone to immune evasion (Fig. 6). We have summarized a potential mechanism of action in Fig. 7, according to which SH42 effectively lowered the membrane cholesterol content (Fig. 1A and B and Fig. S2). This was confirmed by flow cytometry with mCherry-conjugated D4H* to exclusively quantify plasma membrane cholesterol levels in living cells [34,35]. In turn, the reduction in membrane cholesterol led to reduced cholesterol abundance in lipid rafts and manifested in their compromised integrity (Fig. 1C to F), which was reported by a confocal microscopy- and quantitative image analysis-based method [9,36]. These effects were accompanied by lowered cell surface ACE2 levels (Fig. 5A and Fig. S5) that, notably, were of smaller magnitude when compared to reductions of either spike RBD binding or trimer uptake, pointing at the contribution of additional factors. Indeed, we found lower raft localization of ACE2 in response to SH42 (Fig. 5B and C), which may additionally lead to reduced spike binding and uptake. While ATO induced comparable, significant reductions in all of these parameters compared to control samples, SH42 showed an order of magnitude larger inhibitory potency in all examined aspects, which may suggest that SH42 can be a more effective therapeutic alternative for COVID-19. This is further supported by the fact that SH42 reduced cellular infection of replication-competent, complete SARS-CoV-2 virions, and that the effects were significantly larger compared to ATO at a concentration of 10 nM (Fig. 6).

In the current study, we applied a lipophilic statin, ATO, as a reference compound since it requires much lower equivalent doses than other statins, does not need enzymatic activation, and thus can be more reliably used in cellular experiments and it is widely applied in patients [44]. Reduced RBD binding, trimer uptake, and viral replication could be caused by compromised cell viability, a side effect statins are reported to exhibit in certain cell types [44]. Because neither ATO nor SH42 reduced cell viability to any biologically meaningful extent (Fig. S1), we attribute all of the observed phenomena to lowered membrane cholesterol levels and their nontoxic, downstream consequences. Nevertheless, to ensure the comparability of experiments with ATO and SH42, we applied both compounds in our subsequent experiments at 1 μM that can be considered as a dose equivalent to that most commonly applied in cellular studies with statins and 10 nM mimicking statin concentrations that are expected in the serum during conventional treatment protocols [33]. Throughout all of our experiments, SH42 largely outperformed ATO in all aspects in terms of both efficacy and potency supporting its more favorable applicability.

Our findings are in good agreement with previous reports on the role of cholesterol in SARS-CoV-2 infection. Cholesterol essentially influences the function of proteins by determining the biophysical properties and the lateral organization of biological membranes [40,41], and cholesterol-enriched lipid raft microdomains generally serve as entry gates for infectious pathogens [13,20]. The efficiency of SARS-CoV-2 cellular entry is also facilitated by membrane cholesterol at various levels. First, raft partitioning of ACE2 and its colocalization with TMPRSS2 enhances binding and cleavage of the spike [9–11]. Second, SARS-CoV-2 endocytic mechanisms including raft-mediated and clathrin-dependent routes enabling cathepsin L-induced fusion rely on cholesterol [4,5,12,13]. Third, the fusion itself between the viral particle and host membranes is also promoted by cholesterol [45–47].

Consistently, anti-cholesterol and anti-raft approaches were proposed to have potential therapeutic benefits almost from the beginning of the COVID-19 pandemic [13,20]. In accordance with this hypothesis, clinically relevant, membrane cholesterol-depleting cyclodextrins even in drug formulations were shown to reduce cellular binding and entry of SARS-CoV-2 spike glycoproteins, which can provide additional effects to the main antiviral component remdesivir [9], and were found to inhibit replication and release of virus particles, and virus-induced inflammatory response [18] in cellular studies. However, while cyclodextrins may act as active agents in COVID-19, these compounds are mainly applied as drug vehicles [48,49] and rather statins are most commonly used in human medicine to lower cholesterol levels. Statins were indeed found to exert similar anti-SARS-CoV-2 actions in cellular and animal experiments [10,21], and even epidemiological studies suggested their favorable actions in hospitalized COVID-19 patients [22]. On the other hand, other epidemiological studies often failed to demonstrate beneficial statin effects, and thus, their use remains questionable [23], especially in light of their relatively common side effects [24]. DHCR24 inhibitors are novel promising alternatives to block the de novo cholesterol biosynthesis pathway, and their applicability was previously proposed in various pathological conditions including cardiovascular and metabolic disorders, Alzheimer’s disease, prostate cancer, and hepatitis C infection [25]. While several already established compounds were described to inhibit DHCR24 in an off-target manner, and thus with low selectivity and numerous adverse effects, the newly designed SH42 was found to be extremely potent [median inhibitory concentration (IC_50_) = 4.2 nM], highly selective, and nontoxic (IC_50_ > 50 μM). Since SH42 exclusively blocks the terminal desmosterol–cholesterol conversion, it elevates desmosterol and lowers cholesterol levels without affecting the abundance of other proximal sterols on the biosynthetic pathway and, consequently, with a much lower probability of common statin-like side effects such as rhabdomyolysis and liver complications [26]. Furthermore, in cellular and animal models of murine peritonitis and diet-induced human-like nonalcoholic steatohepatitis, SH42 resulted in activation of the liver X receptor α, beneficial metabolic changes with elevated levels of polyunsaturated fatty acids, reduced liver steatosis, and an anti-inflammatory/pro-resolving phenotype [27,28]. Based on the above properties described in literature and our results demonstrating for the first time that SH42 inhibits initial membrane-coupled events of SARS-CoV-2 infection and, consequently, reduces viral RNA copy numbers after cellular infection more efficiently than ATO, it is reasonable to speculate that DHCR24 inhibitors and particularly SH42 may be a more promising novel alternative of cholesterol-lowering agents such as statins and cyclodextrins in COVID-19.

While in our experiments the approximately 40% and 50% reductions in spike RBD binding and trimer entry, respectively, can already be considered at least promising, several lines of argument propose that these actions can even be larger in vivo. (a) As discussed in our previous study in detail [9], the methods applied for Figs. 2 and 4 rely on measurements of fluorescent signals and thus supra(patho)physiological amounts of ligand (spike) and receptors (ACE2), which largely exceed ACE2 expression of in vivo target cells and the amount of spikes on binding virions. However, we previously found that the inhibitory effects of cholesterol reduction become more prominent as the spike protein concentration decreases or, vice versa, a lower extent of cholesterol reduction may induce a relatively larger inhibition in vivo, suggesting that the inhibitory effects of SH42 could in fact be more prominent under therapeutic conditions [9]. In accordance, we observed a negative correlation between the extent of SH42-induced reduction in binding and the applied concentration of RBDs (Fig. 3), raising the possibility of even more pronounced benefits of SH42 in inhibiting early membrane-coupled events of cellular SARS-CoV-2 infection in vivo. (b) Furthermore, when comparing results obtained here using HEK/ACE2 + TMPRSS2 cells with high exogenous ACE2 expression and Calu-3 cells with lower endogenous ACE2 levels, the effects were generally larger in the latter. Therefore, changes induced by SH42 may be even larger in vivo when the numbers of available ACE2 receptors are lower. (c) Drugs and vaccines targeting the spike in a specific manner are prone to reduced efficiency or even resistance due to mutations arising in spike RBD [16,17]. Hence, novel strains may exhibit slight alterations in uptake mechanisms such as observed in Omicron BA.1 that is characterized by much lower reliance on TMPRSS2-mediated entry with a preference for endocytic uptake and thus displaying altered tissue tropism mainly attacking upper respiratory tract epithelia [7,50]. Nevertheless, cellular entry mechanisms seem to remain cholesterol-dependent for all examined variants [5,9,18]. Consistently, in our experiments, SH42 similarly inhibited binding and uptake of variants including WT, Delta, and Omicron BA.1. Furthermore, SH42 effectively reduced copy numbers of viral RNA in the supernatant of cells infected with replication-competent viruses of D614G, Delta, and JN.1 variants (Fig. 6). (d) Although not examined in the current study, based on previously described anti-inflammatory/pro-resolving actions [27,28], it is reasonable to speculate that SH42 can be highly favorable during the aggravated immune response and cytokine storm induced by the SARS-CoV-2 infection. (e) While we only investigated the effects of cholesterol manipulation of the host cell membrane on spike binding and entry, several other membrane-related events, including virion maturation and release [51] and viral membrane fusion with the host cell and consequent syncytia formation [45,47], were shown to be promoted by cholesterol in the host or viral membrane. Consistently, as shown in different viruses, cholesterol may affect fusion between host and virion membranes at various levels such as by affecting membrane curvature generation, molecular ordering, clustering of entry factors, or direct modulation of the formation and stability of entry pores [52,53]. In addition, cholesterol was recently described to affect ACE2–spike interaction in a raft-independent manner as well [54] and enhance membrane binding and embedding of spike even in the absence of ACE2 [55]. These mechanisms may thus represent additional targets of cholesterol-lowering agents in the course of infection.

As a limitation of our study, subsequent steps of cellular infection such as membrane fusion, RNA release and replication, virion assembly, or release were not examined separately. Instead, we applied methods to focus on early membrane-coupled events of SARS-CoV-2 infection, which can be quantified exclusively with our flow cytometry and 3D confocal microscopy-based experimental setups. To examine whether SH42 effects manifest in alterations of the whole lifecycle of the virus and thereby to support the potential therapeutic relevance of our findings, we demonstrated that SH42 effectively reduces copy numbers of the viral RNA in cells infected with complete, replication-competent SARS-CoV-2 virions. The connection between the inhibition of early membrane-coupled steps and reduced cellular infection observed with the replication-competent virus is supported by the decreased entry observed in pseudovirion experiments (Fig. S5). Furthermore, the molecular background of the improved efficiency of SH42 when compared to that of ATO was not elucidated in the study. It can be speculated that SH42, unlike ATO, is devoid of compensatory overexpression and activation of the target enzyme, or, alternatively, elevated desmosterol levels can also participate in beneficial SH42 actions, which would be worth examining in the future. Altogether, our results could lay the foundation for further SH42-based in vivo studies to examine the anti-SARS-CoV-2 potential of the compound.

The most important clinical relevance of our study is that we raised the possibility of the potential role of DHCR24 inhibition by its highly selective and potent blocker SH42 in the adjuvant therapy of COVID-19 by inhibiting initial cholesterol-dependent membrane-coupled steps of cellular SARS-CoV-2 infection. Besides SARS-CoV-2, crucial cholesterol-dependent initial steps of infection, such as binding to a raft-resident cell surface receptor followed by viral uptake, are common mechanisms for various pathogens. For example, receptors of other coronaviruses, namely, human aminopeptidase N for HCoV-229E, ACE2 for HCoV-NL63 and SARS-CoV, and dipeptidyl peptidase 4 for MERS-CoV, localize in these microdomains [56]. Similarly, sialylated glycoprotein and glycolipid influenza receptors [57], or the main human immunodeficiency virus receptor and co-receptors CCR5 and CXCR4 and various glycolipids [58], are primarily found in rafts as well. Furthermore, the glycoprotein of Ebola interacts with cholesterol and the endosomal cholesterol transporter NPC1 to elicit membrane fusion [59,60]. In accordance, cholesterol reduction and raft disruption were proposed a relevant antiviral strategy in these infections [20,57,61], which, based on our findings, potentially points at the more generalized favorable applicability of selective DHCR24 inhibitors such as SH42. Therefore, our experiments may lay the foundation for future in vivo and clinical studies of the clinical application of DHCR24 inhibitors in COVID-19 and other infectious diseases as well.

## Materials and Methods

### Cell cultures and treatments

The human embryonic kidney HEK293T cell line that stably expresses ACE2 and transmembrane serine protease 2 (TMPRSS2) genes (HEK/ACE2 + TMPRSS2) was obtained from GeneCopoeia (Rockville, MD; SL222), while the Calu-3 lung adenocarcinoma cell line with an endogenous expression of ACE2 and TMPRSS2, and the original HEK293T cell line lacking considerable ACE2 and TMPRSS2 were purchased from the American Type Culture Collection (Manassas, VA; HTB-55 and CRL-3216, respectively). Vero cells were obtained from Nuvonis (Vienna, Austria). All cell lines were cultured according to their specifications. For the manipulation of cholesterol levels, HEK/ACE2 + TMPRSS2, Calu-3, Vero, or HEK293T cells were treated for 96 h before the experiments with 10 nM or 1 μM ATO (Sigma-Aldrich, St. Louis, MO; PHR1422) or SH42 (Cayman Chemical, Ann Arbor, MI; 34677) dissolved into the cell culture medium. The concentration of dimethyl sulfoxide (DMSO) used to dissolve ATO and SH42 in stock solutions never exceeded 0.05% during experiments, and pure DMSO was applied at equivalent amount as control.

### Testing cell viability using flow cytometry

The fraction of viable control HEK/ACE2 + TMPRSS2 and Calu-3 cells and those treated for 96 h with 1 or 10 μM ATO or SH42 was determined as described previously [62,63]. Briefly, the supernatant containing cells spontaneously detached from the surface of the cell culture dish was collected and pooled with the adherent cells detached by trypsinization. After washing, this cell suspension was labeled with the necrosis marker Sytox Green Dead Cell Stain (ThermoFisher; S7020) and the apoptosis marker Alexa Fluor 647-conjugated annexin V (ThermoFisher; A23204) applied at dilutions of 1:1,000 and 1:20, respectively, in annexin binding buffer for 15 min at room temperature. Fluorescence intensities of at least 10,000 individual cells per sample were subsequently measured using a NovoCyte 3000RYB flow cytometer (ACEA Biosciences, San Diego, CA). Sytox Green and Alexa Fluor 647 fluorophores were excited at 488 and 640 nm, respectively, and emitted intensities were measured using 530/30-nm and 660/20-nm emission filters, respectively. Subsequently, the fraction of Sytox Green- and annexin V-negative viable cells was calculated for each sample in FCS Express (De Novo Software, Los Angeles, CA), and the fraction of viable cells was calculated as the proportion of double-negative cells.

### Quantification of plasma membrane cholesterol content

To generate a D4H* cholesterol sensor-expressing strain, the *Escherichia coli* BL21 (DE3) strain was transformed with the plasmid pGEX-KG-D4H*-mCherry, a gift from R. Zoncu (Addgene_134604) [64]. The bacteria were grown in Luria broth (LB) until the culture reached an optical density at 600 nm (OD_600_) of approximately 0.6. Protein production was induced with 0.4 mM isopropyl β-d-1-thiogalactopyranoside (IPTG) for 20 h at 18 °C. The bacterial pellet was collected by centrifuging the culture at 2,500*g* for 15 min and then resuspended in Buffer A [20 mM tris–Cl, pH 8.0, 0.1 M NaCl, 1 mM dithiothreitol (DTT)] supplemented with a cOmplete Protease Inhibitor Cocktail tablet (Sigma-Aldrich; CO-RO). The suspension was incubated with 0.35 mg/ml lysozyme (Sigma-Aldrich; L6876) on ice for 30 min. Subsequently, the bacterial lysate was sonicated on ice using Branson Digital Sonifier 450 with 12 cycles of a 10-s pulse followed by a 10-s break, at 50% amplitude output for a total of 4 min. The lysate was treated with 0.5% Triton X-100 and gently rocked at 4 °C for 15 min. Finally, the lysate was clarified by centrifugation at 17,000*g* for 30 min at 4 °C. The clarified supernatant was incubated for 3 h with pre-equilibrated Glutathione Sepharose 4B beads (Sigma-Aldrich; GE17-0756-01) in Buffer A supplemented with 0.1% Triton X-100. The GST-D4H*-mCherry protein was eluted from the beads using a 25 mM l-glutathione solution prepared in 50 mM tris–Cl, pH 8.8, and 200 mM NaCl. The eluate was filtered and concentrated using 10-kDa molecular weight cutoff Amicon Ultra Centrifugal Filter units (Sigma-Aldrich; UFC9010). Further purification was performed using size exclusion high-performance liquid chromatography (SE-HPLC) on a BioBasic SE-1000 column (300 × 7.8 mm, 5 μM particle size, Thermo Scientific; 73605-307846A) with a Shimadzu Prominence HPLC system. SE-HPLC was carried out at a flow rate of 1 ml/min in 50 mM tris buffer, pH 8. Fractions obtained from the SE-HPLC purification were concentrated using Amicon centrifugal filters and stored for subsequent experiments.

For the measurements of plasma membrane cholesterol levels, control HEK293T, Calu-3, or Vero cells and those treated for 96 h with 10 nM or 1 μM ATO or SH42 were trypsinized gently, washed, and subsequently labeled with 5 μM mCherry-conjugated D4H* for 30 min at 37 °C. Fluorescence intensities of at least 10,000 individual cells per sample were subsequently measured using a NovoCyte 3000RYB flow cytometer. mCherry was excited at 561 nm, and the emitted intensity was measured using a 615/20-nm emission filter. During data analysis, the average intensity values were calculated in each sample from data of at least 10,000 cells with a normal morphology on forward scatter (FSC)–side scatter (SSC) dot plots, and subsequently normalized to the mean value determined in untreated control samples.

### Analysis of lateral distribution of membrane cholesterol reduction and lipid raft integrity using confocal laser-scanning microscopy

Control HEK/ACE2 + TMPRSS2 cells grown on 8-well chambered coverglass (ibidi, Grafelfing, Germany; 80826) and those treated for 96 h with 10 nM or 1 μM ATO or SH42 were labeled with 10 nM of the environment-sensitive fluorophore F66 (*N*-[3-(40-dihexylamino-3-hydroxy-flavonyl-6-oxy)-propyl] *N*,*N*-dimethyl-*N*-(3-sulfopropyl)-ammonium inner salt, a kind gift from A. Klymchenko, Université de Strasbourg, Strasbourg, France) and 8 μg/ml Alexa Fluor 647-conjugated CTX (purchased from ThermoFisher, Waltham, MA; C34778) for 20 min on ice. Images were taken from the bottom, flat membrane region of the cells adjacent to the coverglass using an LSM880 confocal laser-scanning microscope (Carl Zeiss AG, Jena, Germany). The F66 dye was excited at 405 nm, and its emission was measured in 2 different wavelength ranges, 463 to 527 nm and 543 to 589 nm, corresponding to the normal (N*) and tautomeric (T*) forms of its excited state, respectively, since its T*/N* fluorescence intensity ratio was previously shown to positively correlate with the cholesterol-dependent magnitude of membrane dipole potential, a molecular order-related membrane biophysical parameter, i.e., higher T*/N* in general represents higher cholesterol abundance [36,38]. Alexa Fluor 647 was excited at 633 nm, and its emission was detected between 649 and 759 nm. During quantitative image analysis carried out with the DipImage toolbox (Delft University of Technology, Delft, Netherlands) under MATLAB (Mathworks, Natick, MA) as described previously [9,36], pixels corresponding to the cell membrane were selected manually and subsequently segmented with a custom-written algorithm in which a threshold intensity was determined for the lipid raft marker Alexa Fluor 647-conjugated CTX using the maxentropy algorithm confirmed by visual inspection. Membrane pixels having Alexa Fluor 647 fluorescence intensities above or below the determined threshold were considered “raft” or “non-raft” pixels, respectively. Then, average T*/N* fluorescence intensity ratios positively correlating with the magnitude of dipole potential were separately calculated from individual pixels in the CTX-high “raft” and CTX-low “non-raft” regions after background subtraction. Considering that the magnitude of dipole potential and thus the F66 T*/N* fluorescence ratio is significantly larger in lipid rafts (*9, 36*), an alternative method was also applied for the analysis. Namely, a threshold value of the T*/N* intensity ratio was determined after comparison of pixelwise intensity ratio histograms of CTX-high and CTX-low regions, and “F66 raft” and “F66 non-raft” pixels were defined as having larger or smaller T*/N* ratio values, respectively, when compared to the threshold. Subsequently, the area fraction of “F66 raft” pixels out of all membrane pixels was calculated for each cell.

### Examination of SARS-CoV-2 spike RBD-GFP binding to ACE2 using flow cytometry

Control HEK/ACE2 + TMPRSS2 Calu-3 and Vero cells, and those treated for 96 h with 10 nM or 1 μM ATO or SH42 were trypsinized, washed, and subsequently incubated in the presence of GFP-tagged WT, Delta, and Omicron BA.1 SARS-CoV-2 spike RBDs (trenzyme, Konstanz, Germany; P2020-031, P2020-049, and P2020-061, respectively). The GFP-RBDs were applied at 0.2 μg/ml in HEK/ACE2 + TMPRSS2 and 1.0 μg/ml in Calu-3 and Vero cells for Fig. 2. For Fig. 3, WT RBD concentrations ranging between 0.1 and 5 μg/ml for HEK/ACE2 + TMPRSS2 and between 1 and 10 μg/ml for Calu-3 cells were utilized. After 4-min incubation, GFP fluorescence intensities were measured in a time window of 30 s with a NovoCyte 3000RYB flow cytometer. GFP was excited at 488 nm, and the emitted intensity was detected using a 530/30-nm emission filter. During data analysis in FCS Express, average intensity values were calculated in each sample from data of at least 10,000 cells with a normal morphology on FSC-SSC dot plots, and subsequently normalized to the mean value determined in untreated control samples.

### Investigation of cellular entry of SARS-CoV-2 spike trimers using confocal laser-scanning microscopy

Control HEK/ACE2 + TMPRSS2, Calu-3, and Vero cells and those treated for 96 h with 10 nM or 1 μM ATO or SH42 were grown on 8-well chambered coverglass. After washing, HEK/ACE2 + TMPRSS2 cells were subsequently incubated for 4 h at 37 °C in the presence of 0.5 μg/ml WT, Delta, or Omicron BA.1 spike trimers conjugated with Alexa Fluor 488 (R&D Systems; AFG10561, AFG10878, and AFG11060, respectively), whereas Calu-3 and Vero cells with 2.5 μg/ml of these trimers. These trimer concentrations were identical in molar concentrations to those used in RBD binding experiments. For the unequivocal visualization of the plasma membrane, cells were stained with 10 nM F66 in the last 15 min of incubation. After washing, 20 to 25 confocal slices were acquired from the cells with a Zeiss LSM880 confocal laser-scanning microscope. F66 and Alexa Fluor 488 were excited at 405 and 488 nm, respectively, whereas their emitted intensities were detected in wavelength ranges of 440 to 480 nm and 493 to 550 nm, respectively. During image analysis, pixels corresponding to the plasma membrane and the intracellular space were identified in F66 Z-stack images using a custom-written 3D watershed algorithm in MATLAB, and the average fluorescence intensity values emitted by Alexa Fluor 488 were calculated using only data of intracellular pixels for each cell. The obtained values were subsequently normalized to the median value determined from data of individual cells in untreated control samples.

### Examination of cell surface ACE2 expression using flow cytometry

Control HEK/ACE2 + TMPRSS2 cells and those treated for 96 h with 10 nM or 1 μM ATO or SH42 were trypsinized and incubated for 30 min in the presence of Alexa Fluor 488-tagged anti-ACE2 antibody (R&D Systems; FAB9332G) applied at a dilution of 1:50. After washing, fluorescence intensities of individual cells were measured with a NovoCyte 3000RYB flow cytometer. Alexa Fluor 488 was excited at 488 nm, and the emitted intensity was detected using a 530/30-nm emission filter. During data analysis in FCS Express, the average intensity values were calculated in each sample from data of at least 10,000 cells with a normal morphology on FSC-SSC dot plots, and subsequently normalized to the mean value determined in untreated control samples.

### Analysis of the colocalization between lipid rafts and ACE2 using confocal laser-scanning microscopy

Control HEK/ACE2 + TMPRSS2 cells grown on 8-well chambered coverglass and those treated for 96 h with 10 nM or 1 μM ATO or SH42 were labeled for 20 min on ice with 8 μg/ml Alexa Fluor 647-conjugated CTX and Alexa Fluor 488-tagged anti-ACE2 antibody applied at a dilution of 1:50. Images were taken from the bottom, flat membrane region of the cells adjacent to the coverglass using an LSM880 confocal laser-scanning microscope. Alexa Fluor 488 and Alexa Fluor 647 were excited at 488 nm and 633 nm, respectively, and their emission was detected in the wavelength ranges of 493 to 540 nm and 650 to 755 nm, respectively. During quantitative image analysis, regions corresponding to membrane pixels were manually selected, and the Pearson correlation coefficient between fluorescence intensities of the 2 applied fluorophores was determined from pixelwise data of the selected regions of individual cells using a custom-written algorithm under MATLAB. To rule out the accidental positive correlation, we determined the 95% confidence interval of the coefficient assuming no correlation between the analyzed parameters for each image according to the method of Costes as described previously [9,36].

### Examination of cellular uptake of WT SARS-CoV-2 spike pseudotyped lentivirions

HEK293T cells were used for the production of SARS-CoV-2 spike protein pseudotyped lentiviral virions. pLenti (a GFP-expressing transfer vector; Addgene, MA, USA), psPAX2 (a packaging plasmid; a kind gift from D. Trono at the University of Geneva Medical School), and pcDNA3.1-SPIKE (Wuhan-Hu-1) (Genscript, NJ, USA) plasmids were used in a 1:1:1 ratio. A day before transfection, HEK293T cells were passaged in order to achieve a 70% confluence the next day (3 × 10^6^ to 5 × 10^6^ cells/T-75 flask). A total of 30 μg of plasmid DNA was used for transfection using polyethyleneimine (PEI) (Sigma-Aldrich, St. Louis, MO, USA). PEI solution was added to the plasmid mixture and incubated for 20 min at room temperature. Next, the medium was removed from the cells and replaced with 5 ml of fresh Dulbecco’s modified Eagle’s medium (DMEM) containing only 1% fetal bovine serum (FBS) without antibiotics. The plasmid mixture was then added to the medium dropwise, and thereafter, the cells were incubated at 37 °C with 5% CO_2_ for 5 h. The medium was then replaced with 10 ml of DMEM containing 10% FBS, 1% penicillin–streptomycin, and 1% glutamine. The supernatant was collected and filtered through a 0.22-μm polyvinylidene fluoride filter (Merck Millipore, Darmstadt, Germany) after 24 and 48 h. The collected supernatants were then pooled together and concentrated by Amicon Ultra centrifugal filter units (Merck Millipore, Darmstadt, Germany). The concentrated viral particles were stored at −70 °C. Enzyme-linked immunosorbent assay (ELISA)-based colorimetric reverse transcriptase (RT) assay (Roche Applied Science, Mannheim, Germany) was used to determine the RT-activity of the pseudovirions according to the manufacturer’s instructions.

Regarding the transduction experiments with pseudovirions, the medium of control HEK/ACE2 + TMPRSS2 cells and those treated for 96 h with 10 nM or 1 μM ATO or SH42, grown in 48-well plates, was changed to 200 μl of fresh medium containing the inhibitors of interest. After a 2-h incubation, Wuhan-Hu-1 spike SARS-CoV-2-based pseudovirions at a concentration of 4 ng/μl RT-equivalent were mixed into DMEM (virus + DMEM free medium = 50 μl) and afterward added to the wells containing the cells. The culture plate was then incubated at 37 °C with 5% CO₂ for 48 h, after which the medium was discarded, and cells were collected in 400 μl of ice-cold phosphate-buffered saline (PBS). Subsequently, fluorescence-activated cell sorting (FACS) analysis (FACSCalibur; BD Biosciences, Singapore) was performed to quantify transduction efficiency by measuring GFP expression. For each sample, 5,000 cells were analyzed and considered positive above a threshold intensity set based on the mock-treated sample. The virus-only control without any pretreatment with ATO or SH42 was set to 100%, and the relative percentage of GFP-positive cells in each experimental condition was calculated with respect to this control.

These experiments were carried out in a biosafety level 2 (BSL-2) laboratory.

### Examination of viral RNA copy numbers in Vero cells infected with replication-competent virions

The D614G (EPI_ISL_1039959), B.1.617.2 (EPI_ISL_17024327), and JN.1 (EPI_ISL_19299615) variants of SARS-CoV-2 were obtained from the strain collection of the National Biosafety Laboratory at the National Center for Public Health and Pharmacy (Hungary). Vero cells were seeded in T25 cell culture flasks (TPP Techno Plastic Products AG, Trasadingen, Switzerland) and maintained in Virus Production Serum-Free Medium (VP-SFM; Gibco, ThermoFisher) supplemented with 2× GlutaMAX-I (Gibco, ThermoFisher). Cells were treated with 10 nM or 1 μM ATO or SH42 for 96 h. Following treatment, cells from each T25 flask were seeded into 96-well plates at a density of 10,000 cells per well. Cells were infected in triplicate with different SARS-CoV-2 variants at 100 TCID_50_ (median tissue culture infectious dose) per well, and cell culture medium containing the corresponding concentrations of ATO or SH42 was used. DMSO treatment served as virus control. After 60 h, 50 μl of supernatant was collected, and RNA was extracted using the Chemagic Total RNA Kit H24 (Revvity, Waltham, MA, USA). SARS-CoV-2 RNA copy numbers were determined by RT-qPCR using the Centers for Disease Control and Prevention (CDC) SARS-CoV-2 N1 primer/probe set on a LightCycler 480 instrument (Roche, Basel, Switzerland) with automated second-derivative analysis and automatic copy number calculation. The copy number control was previously determined using the QC200 AutoDG Droplet Digital PCR System (Bio-Rad, Hercules, CA) with the same primers and probe. These experiments were carried out in a biosafety level 4 (BSL-4) laboratory.

### Statistical analysis

Data obtained with flow cytometry are represented as mean ± SEM from *n* number of independent samples containing data of at least 10,000 cells of normal morphology per sample. In confocal microscopy measurements examining *n* number of cells from at least 5 different experiments, median, first, and third quartile values are plotted in violin plots generated from data of the individual cells. Flow cytometry and trimer uptake data were normalized to the mean and median, respectively, of untreated control samples, which was required to reduce within-group variability due to differences between initial fluorescence intensities, which would have obscured between-group differences. Outliers were included in data analysis and presentation. Statistical analysis was carried out in GraphPad Prism (La Jolla, CA). Multiple comparisons were performed with one-way analysis of variance (ANOVA), and *P* values were calculated based on post hoc Tukey’s honestly significant difference (HSD) tests that were conducted only if the data were normally distributed and *F* in ANOVA achieved *P* < 0.05. Differences were considered significant when *P* < 0.05 (*P < 0.05, **P < 0.01, ***P < 0.001, and ****P < 0.0001).

## Data Availability

Experimental data supporting the conclusions of this study are available within the article and the Supplementary Materials. All other data supporting the findings of this study are available from the corresponding author upon reasonable request.
