# Supplementary material for: The Selective DHCR24 Blocker SH42 Inhibits ACE2 Binding and Cellular Entry of SARS-CoV-2 Spike Proteins More Efficiently Than Atorvastatin
Source: Research (Wash D C). 2026 May 14;9:1280. doi: 10.34133/research.1280 (PMC13172576; doi:10.34133/research.1280)
Supplement: Supplementary 1 — Figs. S1 to S6 [file research.1280.f1.zip › Kovacs et al_suppl_re-rev_anonymus highlighted.docx]

Supplementary Materials for

**The selective DHCR24 blocker SH42 inhibits ACE2 binding and cellular entry of SARS-CoV-2 spike proteins more efficiently than atorvastatin**

**This PDF file includes:**

Figs. S1 to S6


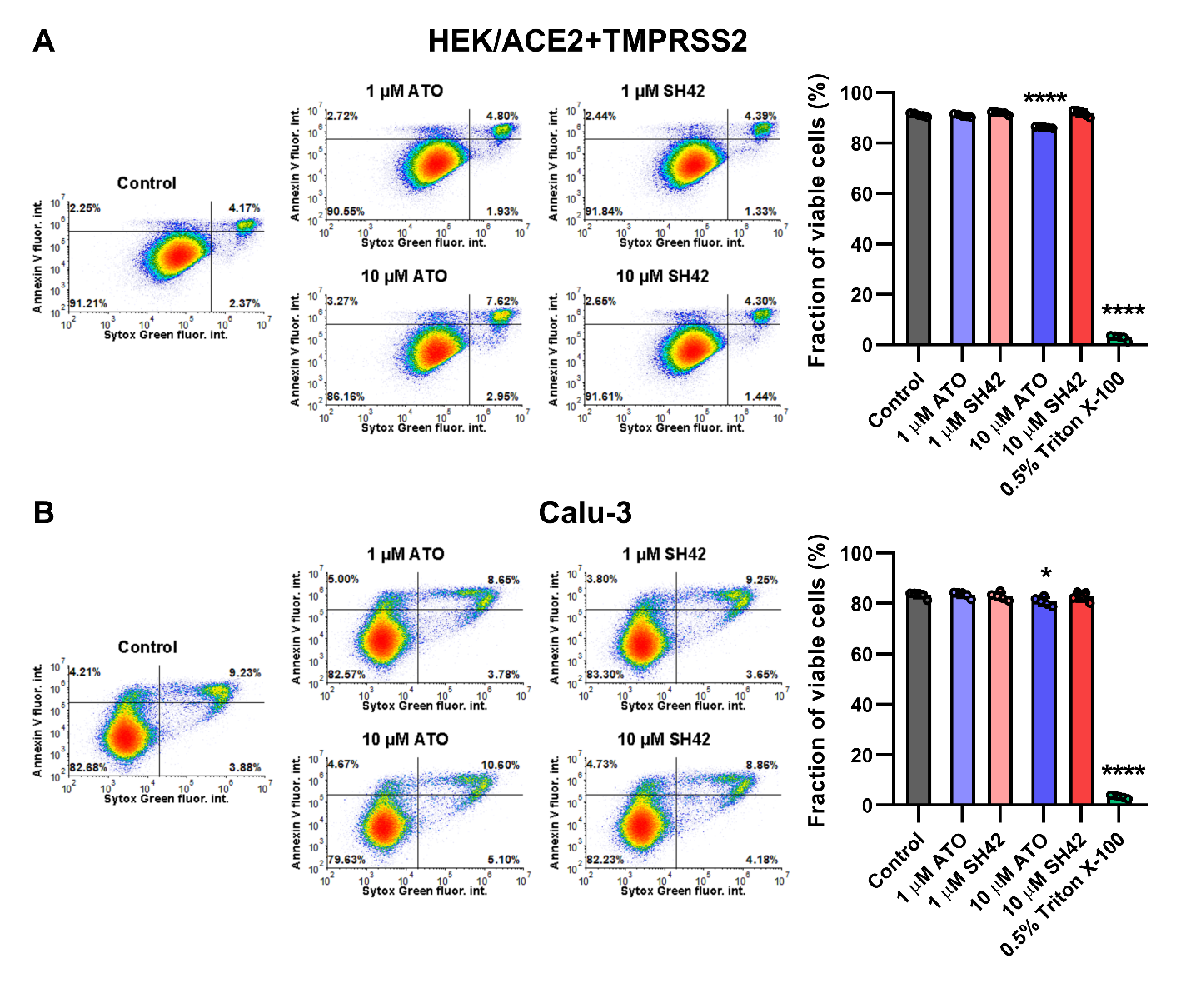


Fig. S1. The effects of atorvastatin and SH42 on cell viability

HEK/ACE2+TMPRSS2 (A) and Calu-3 (B) cells were treated for 96 h with 1 or 10 µM atorvastatin (ATO) or SH-42, or, as a positive control, for 1 h with 0,5% Triton X-100. After pooling cells in suspension and those detached by trypsinization, the cells were labeled with Sytox Green and Alexa Fluor 647-conjugated annexin V to identify necrotic and apoptotic cells, respectively. Fluorescence intensities of individual cells were measured using flow cytometry and the relative fraction of double negative viable cells was determined in each sample containing at least 10,000 cells. Representative density plots demonstrate the effects of ATO and SH42 on cell viabilities. The viable ratios obtained in n = 5 biological replicates, and their average values (± SEM) are plotted in the figure. Asterisks indicate significant differences compared to control samples (*p < 0.05, ****p < 0.0001), which were determined by Tukey’s HSD test carried out after significant differences were obtained for between-group effects in ANOVA.


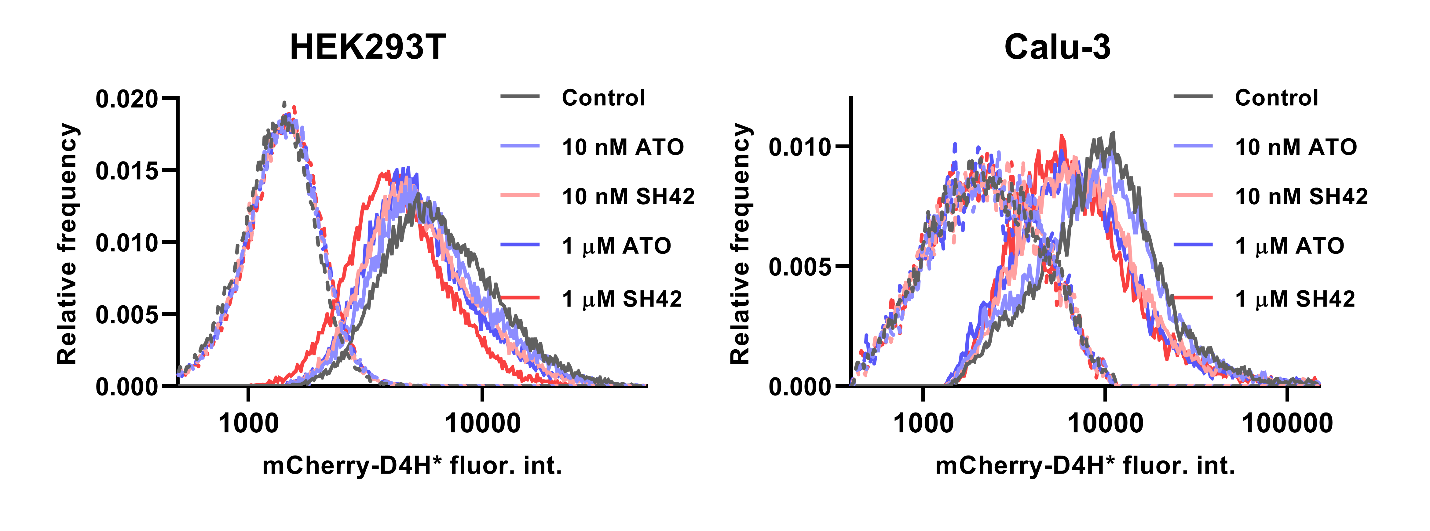


Fig. S2. Representative fluorescence intensity histograms demonstrating reduced cellular D4H* binding correlating with decreased plasma membrane cholesterol levels in response to atorvastatin and SH42

Control HEK293T (A) and Calu-3 (B) cells and those treated for 96 h with 10 nM or 1 µM atorvastatin (ATO) or SH-42 were labeled with mCherry-conjugated D4H*, the D434S mutant of domain four (D4) of the cholesterol binding *Clostridium perfringens* theta-toxin. Representative fluorescence intensity histograms of at least 10,000 individual cells of normal morphology per sample examined with flow cytometry demonstrate the extents of D4H* binding when compared to unlabeled cells (dashed lines), and the effects of ATO and SH42 on D4H* fluorescence intensities positively correlating with plasma membrane cholesterol abundance.


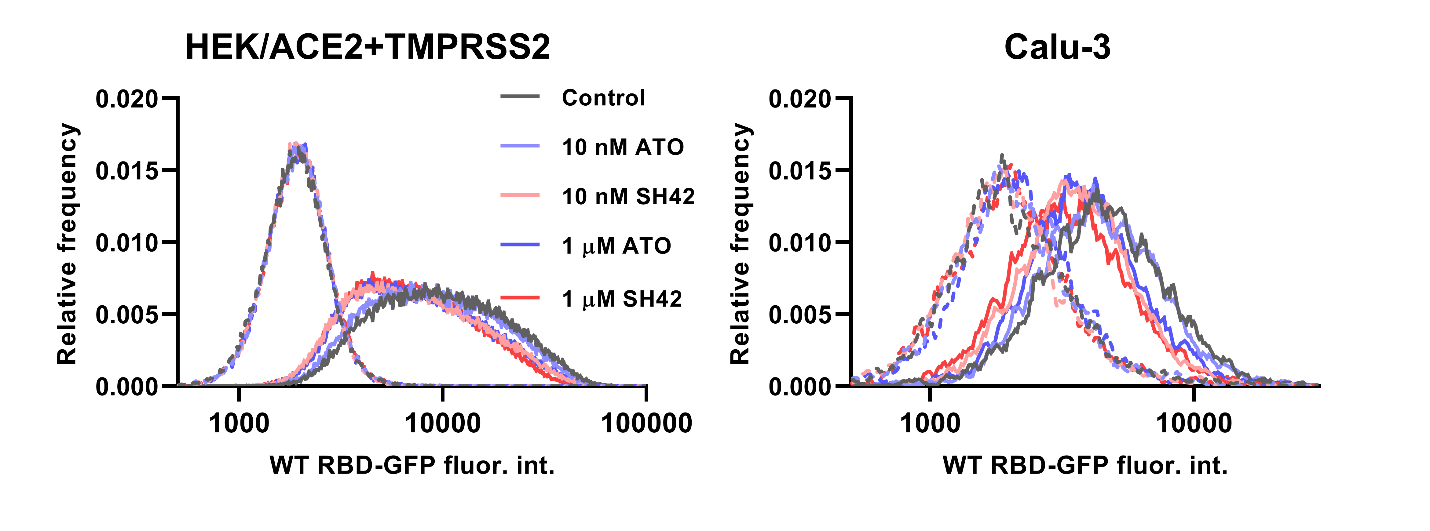


Fig. S3. Representative fluorescence intensity histograms demonstrating reduced ACE2 binding of GFP-conjugated SARS-CoV-2 spike receptor-binding domains in response to atorvastatin and SH42

Control HEK/ACE2+TMPRSS2 (A) and Calu-3 (B) cells and those treated for 96 h with 10 nM or 1 µM atorvastatin (ATO) or SH-42 were incubated in the presence of Wuhan-Hu-1 strain of SARS-CoV-2 spike receptor-binding domains conjugated with GFP (WT RBD-GFP) for 4 min. Representative fluorescence intensity histograms of at least 10,000 individual cells of normal morphology per sample examined with flow cytometry demonstrate the extents of WT RBD-GFP binding when compared to unlabeled cells (dashed lines), and the effects of ATO and SH42 on WT RBD-GFP binding.


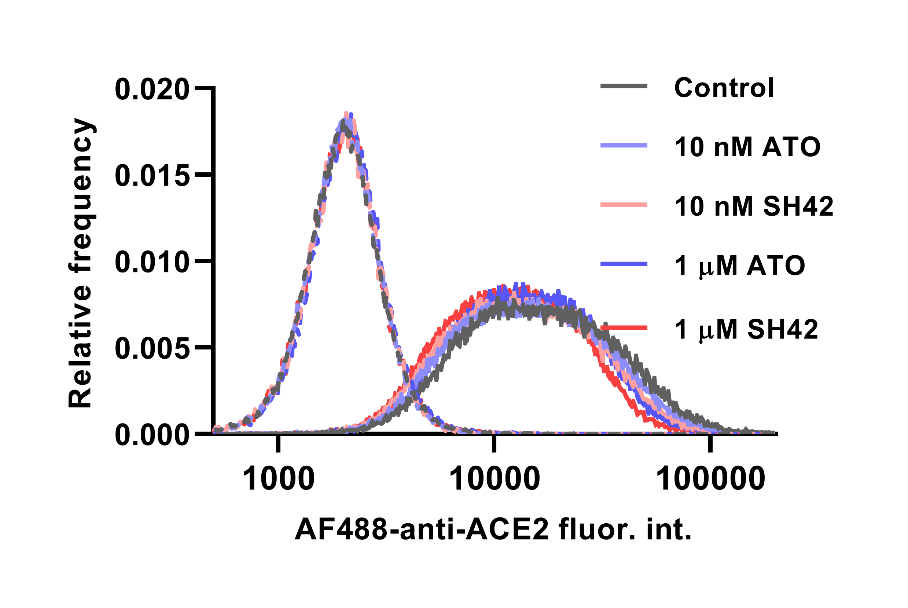


Fig. S4. Representative fluorescence intensity histograms demonstrating reduced cell surface ACE2 expression in response to atorvastatin and SH42

The levels of cell surface ACE2 of control HEK/ACE2+TMPRSS2 cells and those treated for 96 h with 10 nM or 1 µM atorvastatin (ATO) or SH-42 were examined using Alexa Fluor 488-conjugated anti-ACE2 antibodies (AF488-anti-ACE2) and flow cytometry. Representative fluorescence intensity histograms of at least 10,000 individual cells of normal morphology per sample examined with flow cytometry demonstrate the extents of AF488-anti-ACE2 binding when compared to unlabeled cells (dashed lines), and the effects of ATO and SH42 on AF488-anti-ACE2 binding positively correlating with plasma membrane ACE2 abundance.


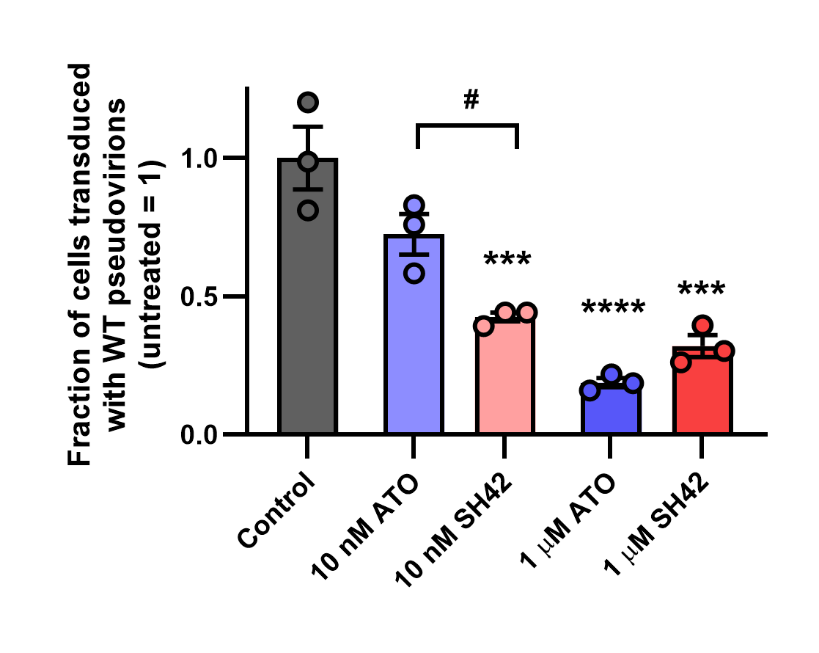


Fig. S5. SH42 and atorvastatin reduce cellular uptake of WT SARS-CoV-2 spike pseudotyped lentivirions

Control HEK/ACE2+TMPRSS2 cells and those treated for 96 h with 10 nM or 1 µM ATO or SH42 were transduced with Wuhan-Hu-1 (WT) spike SARS-CoV-2-based pseudovirions at a concentration of 4 ng/µl RT-equivalent for 48 h in the presence or absence of 10 nM or 1 µM ATO or SH42. After discarding the medium, cells were collected and their transduction efficiency was quantified by measuring GFP expression. For each sample, 5,000 cells were analyzed and considered positive above a threshold intensity set based on the mock-treated sample. The relative number of positive cells were normalized to the average of virus-only controls. The normalized fraction of positive cells obtained in n = 3 independent biological replicates, and their average values (± SEM) are plotted in the figure. Asterisks indicate significant differences compared to control samples (***p < 0.001, ****p < 0.0001), while hashes show those between samples treated with ATO and SH42 at identical concentrations (^#^p < 0.05), which were determined by Tukey’s HSD test carried out after significant differences were obtained for between-group effects in ANOVA.


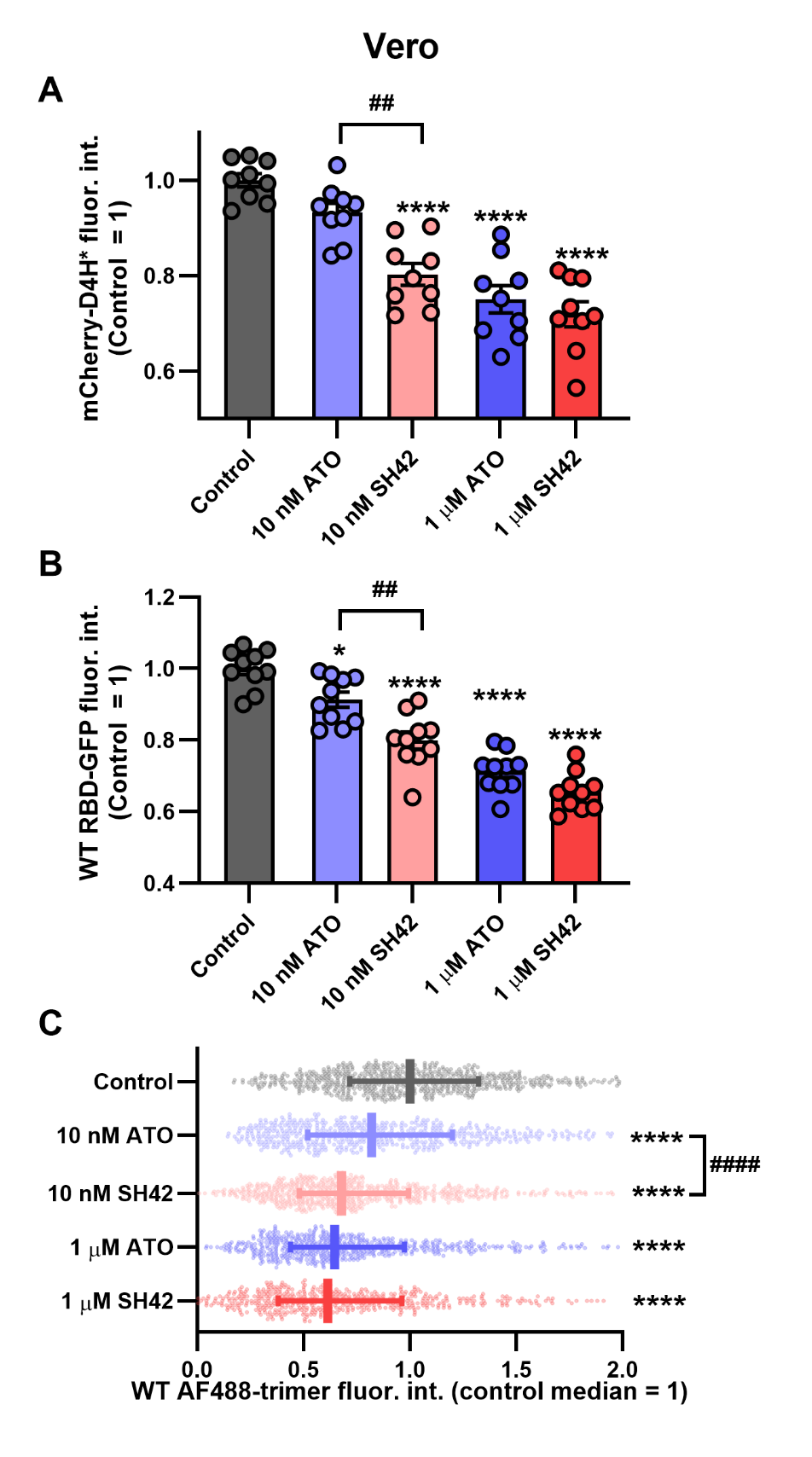


Fig. S6. SH42 reduces plasma membrane cholesterol abundance, ACE2 binding of WT SARS-CoV-2 spike receptor-binding domains and the entry of WT SARS-CoV-2 spike trimers more efficiently than atorvastatin in Vero cells

(A) Control Vero cells and those treated for 96 h with 10 nM or 1 µM atorvastatin (ATO) or SH42 were labeled with cholesterol-binding mCherry-conjugated D4H*, the D434S mutant of domain four (D4) of *Clostridium perfringens* theta-toxin. Fluorescence intensities correlating with plasma membrane cholesterol levels of at least 10,000 individual cells per sample were subsequently measured using a flow cytometer. The average intensity values obtained in n = 10 independent biological replicates, and their average values (± SEM) are plotted in the panel. (B) Vero cells treated as above were incubated with GFP-conjugated Wuhan-Hu-1 (WT) receptor-binding domains (RBDs) for 4 min. Fluorescence intensities of at least 10,000 individual cells per sample were subsequently measured using a flow cytometer. The average intensities obtained in n = 10 independent biological replicates, normalized to the mean value determined in untreated control samples, along with their average values (± SEM) are plotted in the panel. (C) Vero cells treated as above were incubated for 4 h in the presence of WT SARS-CoV-2 spike trimers conjugated with Alexa Fluor 488 (AF488-trimers) and labeled with F66. During image analysis, pixels corresponding to plasma membrane and intracellular pixels were segmented based on F66 Z-stack images. Subsequently, the average fluorescence intensity values emitted by AF488-trimers were calculated exclusively from data of intracellular pixels for individual cells. The average intensities obtained in n = 600-700 cells and normalized to the median value determined in untreated control samples are plotted along with median values with quartiles. Asterisks indicate significant differences compared to control samples (*p < 0.05, ****p < 0.0001), while hashes show those between samples treated with ATO and SH42 at identical concentrations (^##^p < 0.01, ^####^p < 0.0001), which were determined by Tukey’s HSD test carried out after significant differences were obtained for between-group effects in AN
