# Supplementary figures and images for: The Selective DHCR24 Blocker SH42 Inhibits ACE2 Binding and Cellular Entry of SARS-CoV-2 Spike Proteins More Efficiently Than Atorvastatin
Source: Research (Wash D C). 2026 May 14;9:1280. doi: 10.34133/research.1280 (PMC13172576; doi:10.34133/research.1280)

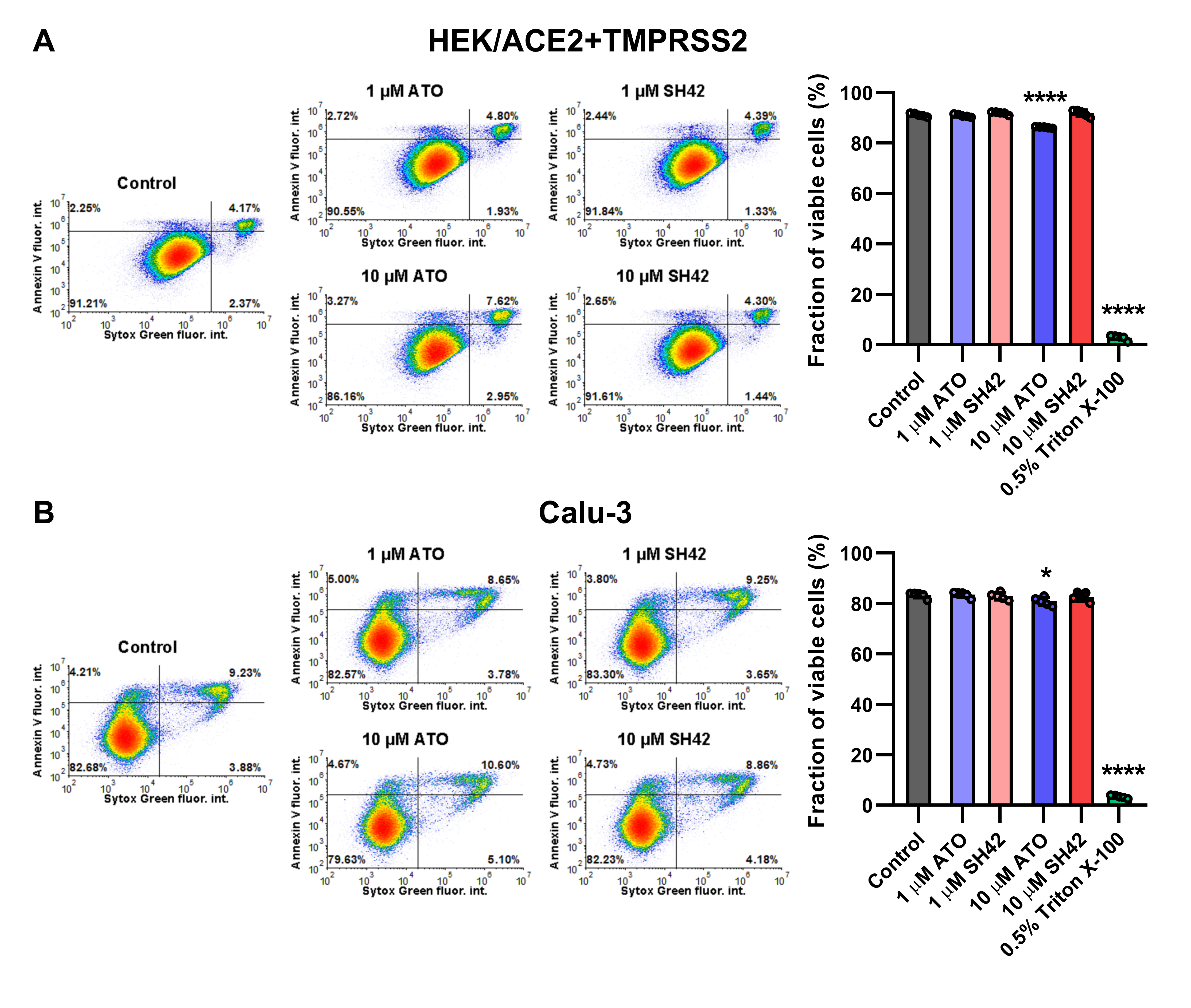

Supplement: Supplementary 1 — Figs. S1 to S6 [file research.1280.f1.zip › Fig S1.tif]

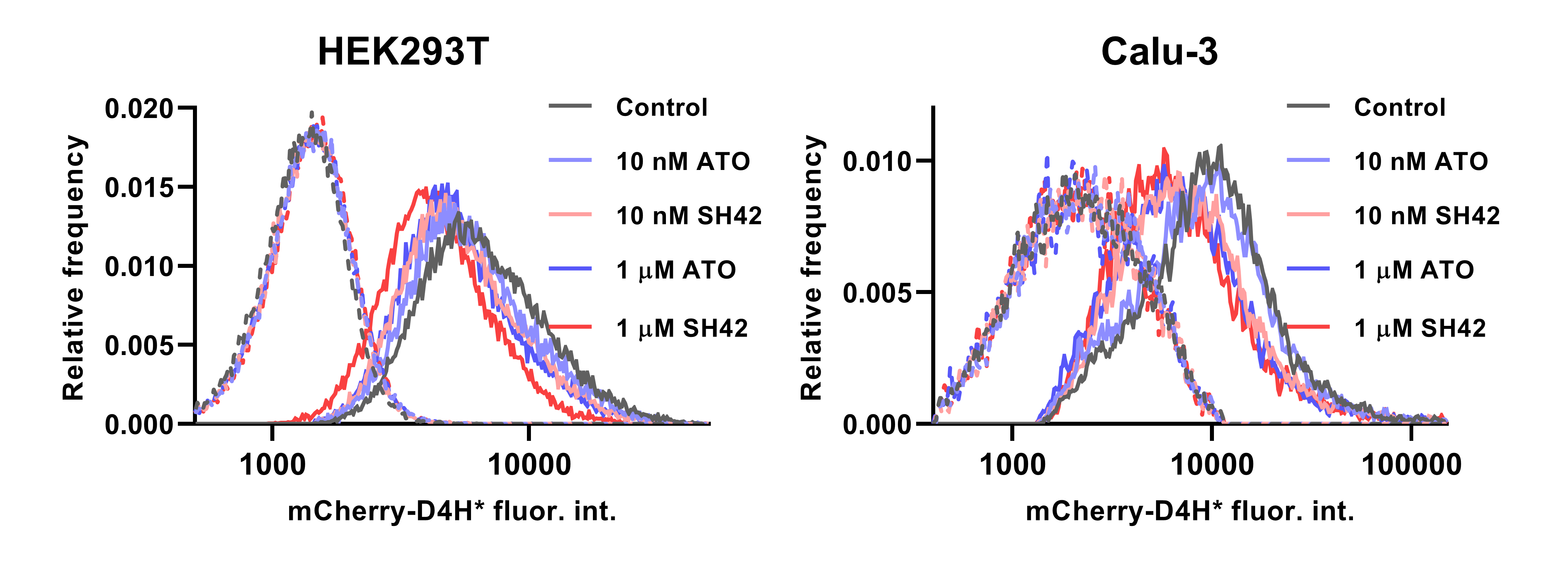

Supplement: Supplementary 1 — Figs. S1 to S6 [file research.1280.f1.zip › Fig S2.tif]

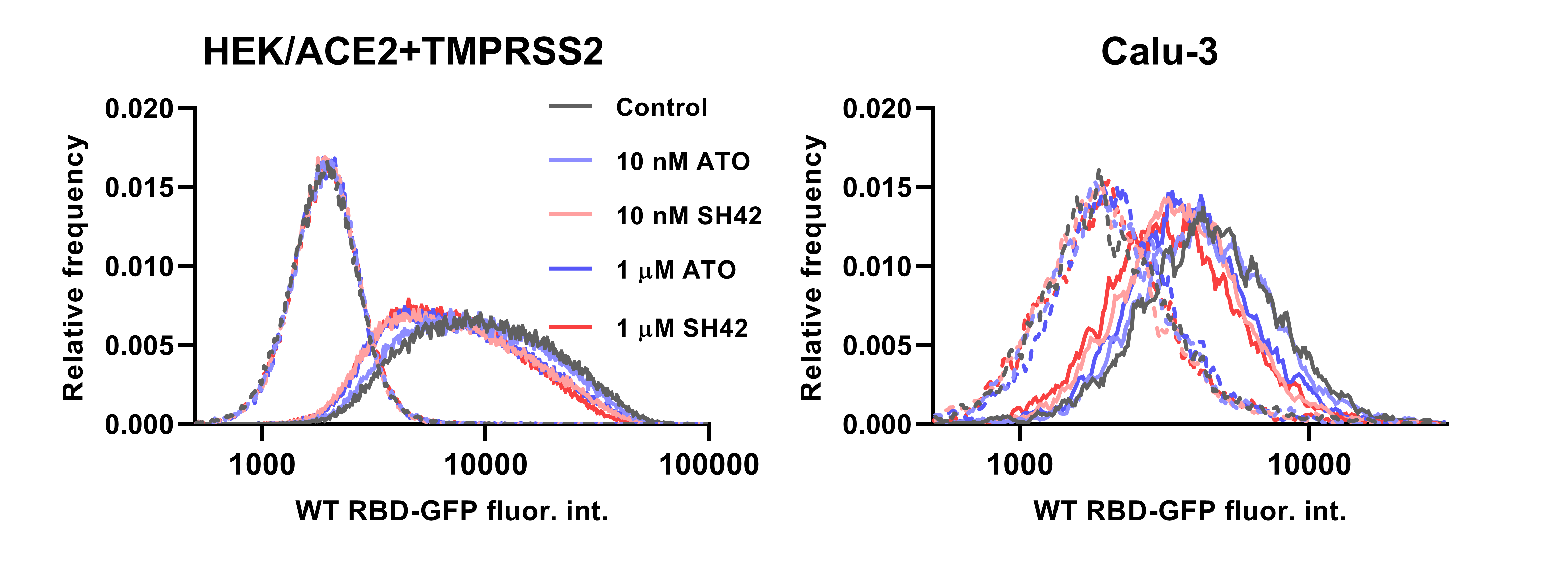

Supplement: Supplementary 1 — Figs. S1 to S6 [file research.1280.f1.zip › Fig S3.tif]

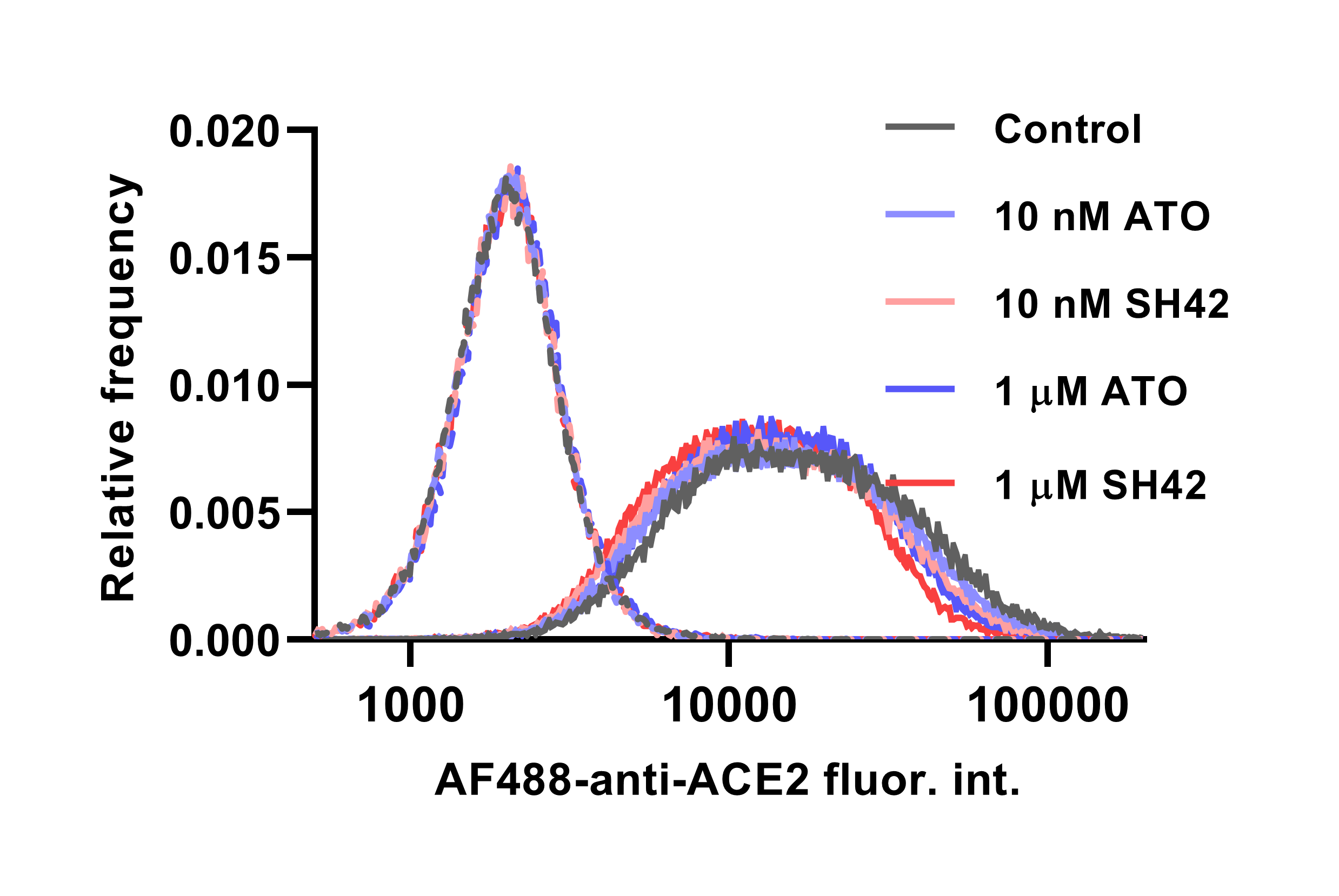

Supplement: Supplementary 1 — Figs. S1 to S6 [file research.1280.f1.zip › Fig S4.tif]

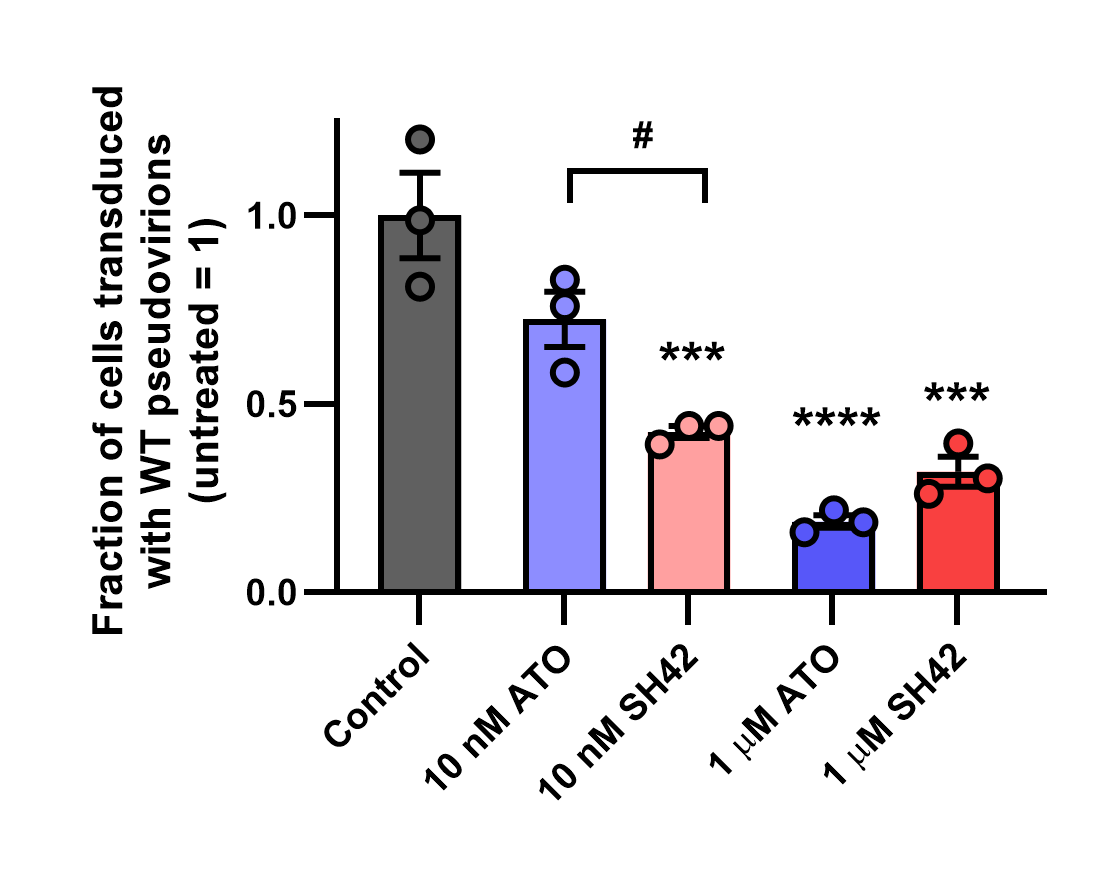

Supplement: Supplementary 1 — Figs. S1 to S6 [file research.1280.f1.zip › Fig S5.tif]

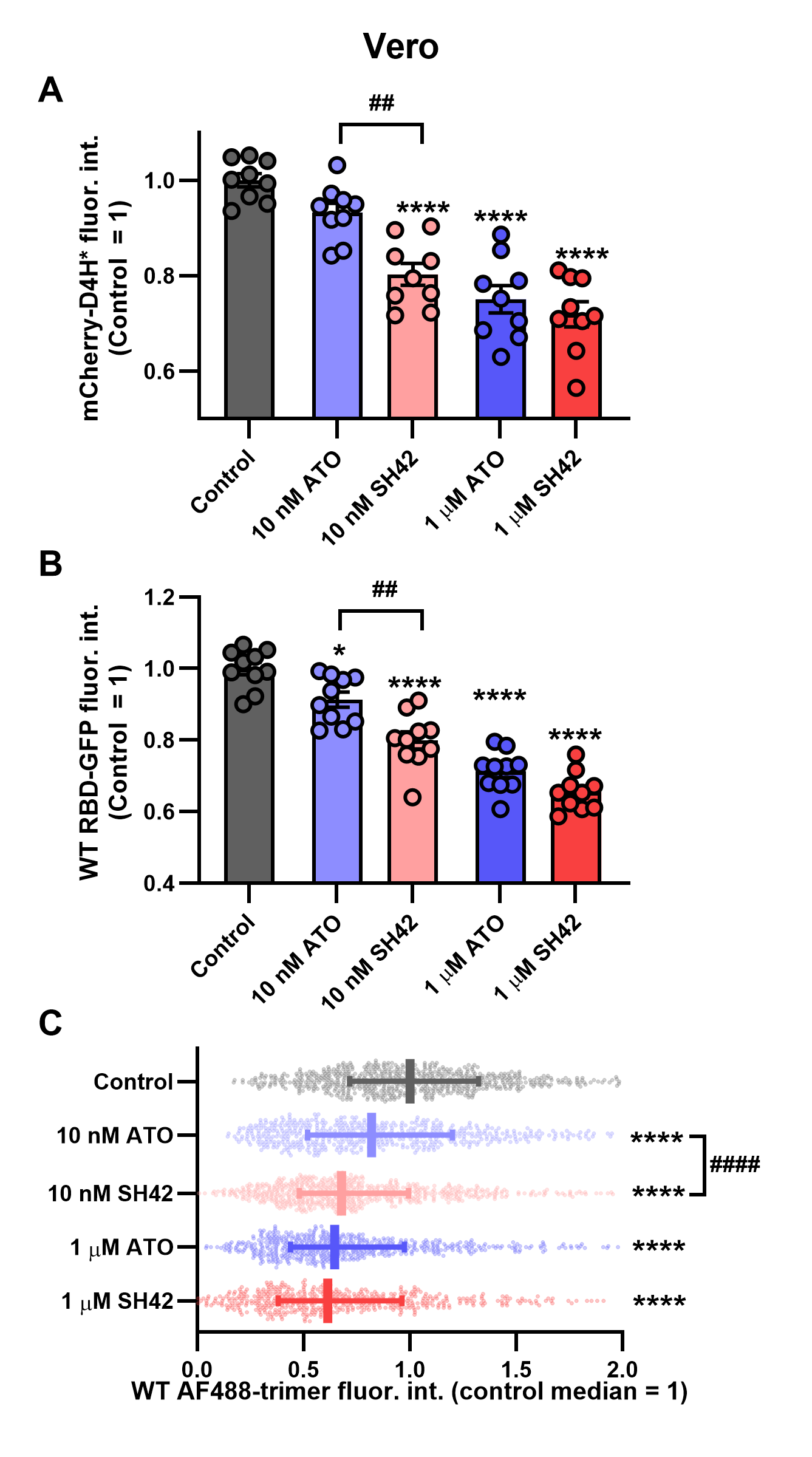

Supplement: Supplementary 1 — Figs. S1 to S6 [file research.1280.f1.zip › Fig S6.tif]
